# Strong and widespread action of site-specific positive selection in the snake venom Kunitz/BPTI protein family

**DOI:** 10.1038/srep37054

**Published:** 2016-11-14

**Authors:** Vera Župunski, Dušan Kordiš

**Affiliations:** 1 Department of Chemistry and Biochemistry, Faculty of Chemistry and Chemical Technology, University of Ljubljana, Ljubljana, Slovenia; 2 Department of Molecular and Biomedical Sciences, Josef Stefan Institute, Ljubljana, Slovenia

## Abstract

S1 family of serine peptidases is the largest family of peptidases. They are specifically inhibited by the Kunitz/BPTI inhibitors. Kunitz domain is characterized by the compact 3D structure with the most important inhibitory loops for the inhibition of S1 peptidases. In the present study we analysed the action of site-specific positive selection and its impact on the structurally and functionally important parts of the snake venom Kunitz/BPTI family of proteins. By using numerous models we demonstrated the presence of large numbers of site-specific positively selected sites that can reach between 30–50% of the Kunitz domain. The mapping of the positively selected sites on the 3D model of Kunitz/BPTI inhibitors has shown that these sites are located in the inhibitory loops 1 and 2, but also in the Kunitz scaffold. Amino acid replacements have been found exclusively on the surface, and the vast majority of replacements are causing the change of the charge. The consequence of these replacements is the change in the electrostatic potential on the surface of the Kunitz/BPTI proteins that may play an important role in the precise targeting of these inhibitors into the active site of S1 family of serine peptidases.

Kunitz/BPTI family (or Inhibitor family I2 in Merops Db (http://merops.sanger.ac.uk/)) comprise inhibitors of serine peptidases that belong to the family S1 and include trypsin, chymotrypsin, tissue kallikrein and plasmin^1^. As evident from the Merops Db Kunitz/BPTI family is distributed mainly in metazoans. Proteins in the Kunitz/BPTI family can have single or multiple Kunitz domains linked together, or are associated with other domain types^2^,^3^. The Kunitz domain comprises around 60 amino acid residues stabilized by three disulfide bonds. The inhibitory specificity of the Kunitz domain varies with the particular amino acids at the reactive sites and exhibit canonical inhibition^4^.

Kunitz/BPTI inhibitors are small proteins (around 6 kDa) with a compact structure composed of a hydrophobic core, containing a central β-sheet and three disulfide bridges with conserved chiralities^5^,^6^. This core is the scaffold that supports the convex and exposed canonical binding loop at positions P3–P3′, according to the Pn–Pn′ notation of Schechter and Berger^7^. This loop is highly complementary to the concave protease active site and is, thus, responsible for the extreme stability of the interaction with the target enzyme in a substrate-like manner^8^. The dissociation constants of enzyme-inhibitor complexes range from 10^−13^ to 10^−7^ M, mainly depending on the nature of the residue at position P1 and the number of contacts formed with the S1 site of the bound protease^5^. Typically, trypsin inhibitors contain Arg/Lys at position P1, whereas chymotrypsin inhibitors have Leu/Met at this position. However, other residues at the primary binding site and within the secondary binding loop (residues 34–39 in BPTI) are also suggested to have significant influence on the association energy^5^. The common interaction pattern of canonical Kunitz/BPTI inhibitors remained largely conserved throughout the evolution from invertebrates to mammals, significantly depending on the P2, P1, and P1′ residues of the inhibitor^6^.

The vast majority of snake Kunitz/BPTI inhibitors inhibits trypsin or chymotrypsin, and some variants exhibit specific affinity for trypsin or chymotrypsin^9^,^10^. Potent inhibition of trypsin has been attributed to the presence of a charged lysine residue at the P3′ position. Functional derivatives include plasmin inhibitors, potassium-channel blockers and calcium-channel blockers^11^,^12^,^13^. Structural derivatives have roles that include disulfide-linked subunits of toxin complexes such as β-bungarotoxin from *Bungarus* and taicatoxin from *Oxyuranus* venoms^14^. Despite significant overall sequence variation, the Kunitz/BPTI toxins share a motif in which the minimum ion-channel pharmacophore is defined by a critical basic residue (usually a lysine) located 7 Å from a hydrophobic residue. This functional site, assisted by various constellations of “secondary” residues for K+ channel binding, is quite distinct from the protease inhibition site^12^,^13^,^14^. The same fold is therefore being used for two distinct functions, but different side chains of the molecule are involved in each^15^.

Ancestral function of Kunitz/BPTI proteins is the inhibition of S1 family of serine peptidases^15^,^16^. In a number of organisms a secondary loss of inhibitory activity occurred and novel functions originated, such as the potassium-channel and calcium-channel blockers^15^,^17^. These novel functions originated independently several times in diverse animal phyla, such as cnidarians, arachnids and in some snakes^15^. Recently, some snake Kunitz/BPTI proteins were found to be bifunctional inhibitors, since they inhibit serine peptidases and act as potassium-channel blockers^18^. Gene structures of Kunitz/BPTI genes are known for a number of organisms, single domain containing Kunitz/BPTI genes possess highly conserved organization of 3 exons and 2 introns^19^,^20^,^21^,^22^,^23^. In some snakes a WAP domain can be present in Kunitz/BPTI genes forming a KU-WAP proteins^23^.

Evolutionary studies of Kunitz/BPTI family have been made in diverse organisms, in snakes^10^, ticks^24^ and in vampire bat^25^. We were the first to demonstrate adaptive evolution in snake Kunitz/BPTI family^10^. At that time the amount of the available snake cDNAs was very limited. In the last years the number of snake transcriptomes and genomes has tremendously increased. This prompted us to reanalyse molecular evolution of Kunitz/BPTI family in snakes with the latest codon-based models to infer the action of site-specific positive selection on much larger sample of snake Kunitz/BPTI genes and cDNAs.

The goal of this study was to assess the selection patterns and evolutionary history of the Kunitz/BPTI genes in Viperidae snakes at the gene and protein level. We obtained numerous novel Kunitz/BPTI genes and cDNAs from the available venomous snake genomes and transcriptomes. The quality of available venomous snake draft genomes is still not very high, causing the problem in obtaining the whole length Kunitz/BPTI genes or larger genomic contigs that contain tandemly organized Kunitz/BPTI genes. The dataset we used to infer the action of site-specific positive selection in snake Kunitz/BPTI genes and cDNAs was large, reaching 100 sequences. One drawback with gene-wide Ka/Ks analyses is that they might miss signals of adaptive sequence evolution that are restricted to certain parts of a gene only^26^. To account for this, we analyzed our gene set using Selecton^27^,^28^ and Datamonkey^29^ servers to infer site-specific selection pressures acting on snake Kunitz/BPTI genes, and correlated the results with known structural and experimental data. As expected, we found strong evidence for positive selection acting on the snake Kunitz/BPTI genes, demonstrating that adaptive evolution was a force driving their evolution.

## Results and Discussion

### Snake venom Kunitz/BPTIs are encoded by multigene families

By screening of *Vipera ammodytes* genomic library we isolated four different Kunitz/BPTI genes. Five additional Kunitz/BPTI genes were obtained by PCR amplification of *V. ammodytes* genomic library. The lengths of the PCR amplified genes were 1449–1456 bp, while more than 2 kb of genomic clones have been sequenced. Seven *V. ammodytes* Kunitz/BPTI genes are chymotrypsin inhibitors since P1 amino acid residue is leucine (L), while two genes (clones 562E and 648) encode trypsin inhibitors since a typical lysine (K) is located at the P1 site^8^ (Suppl. Fig. 1).

**Figure 1:**
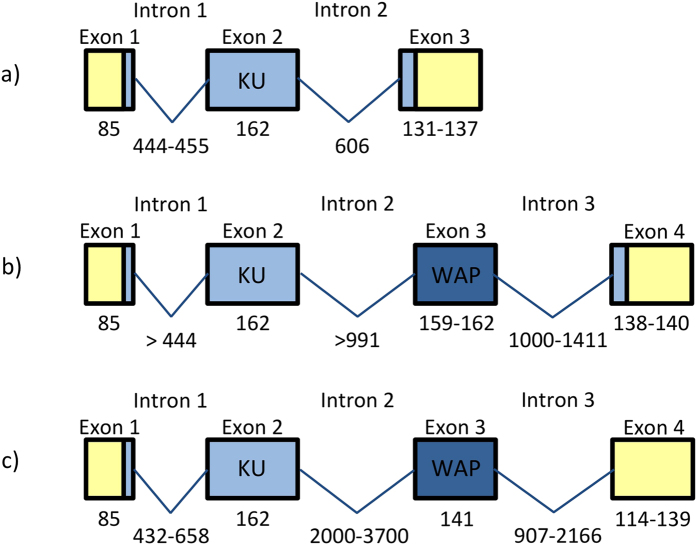
Structural organization of the snake venom Kunitz/BPTI genes. (**a)** Structure of the *Vipera ammodytes* Kunitz/BPTI genes with 3 exons and 2 introns. (**b)** Structure of the *Vipera berus* and *Ophiophagus hannah* KU-WAP genes. WAP exon is inserted into the second intron of the Kunitz/BPTI genes. Introns have different lengths. (**c)** Two *Ophiophagus* and two *Vipera berus* KU-WAP genes have stop codons at the end of the exon 3, therefore the conserved sequence of the exon 4 is noncoding 3′UTR (see Suppl. Table 1). Exons are represented as boxes: 5′UTR and 3′UTR are in yellow, Kunitz coding sequences are in light blue, WAP coding sequences are in dark blue, introns are represented as connecting lines.

To determine the gene structure of *V. ammodytes* Kunitz/BPTI genes, we first obtained the sequence of genomic DNA. A comparison of the *V. ammodytes* Kunitz/BPTI genomic sequence with the corresponding cDNA sequences^10^ revealed that the genes contain three exons (a 5′ exon, an internal exon, and a 3′ exon) interrupted by two introns, which possess canonical GT-AG splice sites. First exon encodes signal peptide and 4 amino acid residues of a propeptide, major part of the protein is encoded in the second exon, while the remaining 8–13 amino acid residues are encoded in the third exon together with the 3′UTR (Fig. 1, Suppl. Fig. 2). *V. ammodytes* Kunitz/BPTI genes possess the same exon-intron organization as the other known snake Kunitz/BPTI genes^19^,^20^,^21^,^22^,^23^ (Suppl. Table 1). The lengths of exons in all *V. ammodytes* Kunitz/BPTI genes range in size from 85 to 162 bp. The first and the third exon are the most conserved exons of all Viperidae Kunitz/BPTI genes. Both introns of the *V. ammodytes* Kunitz/BPTI genes are located in positions homologous to those occupied by the introns of the snake Kunitz/BPTI genes^19^,^20^,^21^,^22^,^23^ (Suppl. Table 1). In Kunitz/BPTI genes of venomous snakes, the comparison of the sizes and nucleotide sequences of all introns shows a very high level of conservation (Suppl. Table 1). The CR1 LINE element is present in the second intron of *V. ammodytes* Kunitz/BPTI genes.

New snake venom Kunitz/BPTI genes have been searched in different WGS databases. In draft genomes of *Vipera berus, Crotalus mitchellii* and *Ophiophagus hannah* we have found numerous novel genes with Kunitz domain. Some of them contain single Kunitz domain while others encoded both Kunitz and WAP domains. Since the genome of *C. mitchellii* contain quite short contigs, we have obtained only four partial genes with Kunitz domain. In draft genomes of *V. berus* and *O. hannah* we have found several full length Kunitz/BPTI genes and Kunitz-WAP genes (Suppl. Table 1).

KU-WAP genes contain 4 exons and 3 introns. It is apparent that the exon encoding WAP domain is inserted into the intron 2 of Kunitz/BPTI gene (Fig. 1). Interestingly, exons 1 and 2 and intron 1 of Kunitz/BPTI genes are nearly the same in KU-WAP genes as well as the last exons (exons 3 and 4, respectively). Kunitz/BPTI and KU-WAP genes have highly similar sequences and gene organization. In contrast to the Kunitz/BPTI genes, the introns 2 and 3 of KU-WAP genes are longer, with some sequence similarities of intron 2 from Kunitz/BPTI genes. The lengths of introns 2 and 3 are quite variable (Suppl. Table 1).

### Tandem duplications of Kunitz/BPTI MFs in Viperidae snakes–evidence from *V. berus* draft genome

The availability of a few snake draft genomes allowed us to examine not only gene sequences but also the locations and orientations of Kunitz/BPTI and KU-WAP genes in Viperidae snake genomes. We found that *V. berus* possess three KU-WAP genes within a single contig (accession number JTGP01134455, size of this contig is 19,647 bp). The presence of three KU-WAP genes in the same genomic contig is highly suggestive of intrachromosomal gene duplications. These three KU-WAP paralogs in the *V. berus* genome occur next to one another in a head-to-tail orientation.

**Figure 2:**
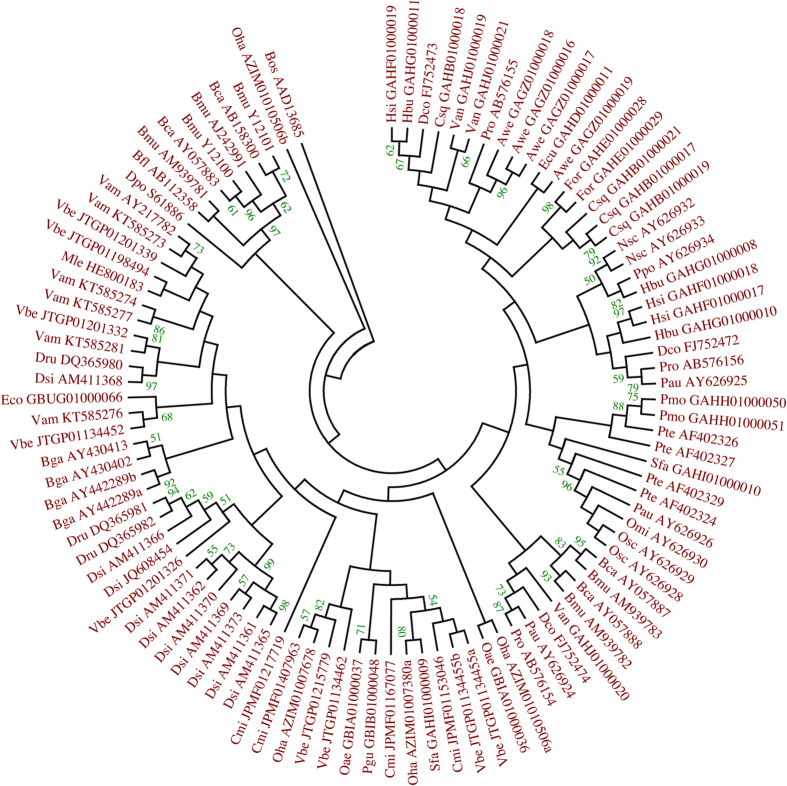
ML phylogeny of the snake venom Kunitz/BPTIs. Phylogenetic analysis of the snake venom Kunitz/BPTI genes was conducted using the translated amino acid sequences of the exon 2. We used MEGA6^53^ to select the best model of sequence evolution for the snake venom Kunitz/BPTI family. The best fit model of amino acid substitution for our data set was determined as JTT + G. ML tree represents the bootstrap consensus following 500 replicates, nodes with confidence values greater than 50% are indicated. As an outgroup, we used bovine BPTI. Phylogenetic analyses were performed with the program MEGA6^53^.

The only data on tandem duplications of Kunitz/BPTI genes are available from the ticks^24^, where the authors found 46 dispersed genes and 9 tandemly duplicated genes. However, the latest tick (*Ixodes scapularis*) genome data available on Ensemble (http://metazoa.ensembl.org/Ixodes_scapularis/Info/Index) revealed the opposite, with tandem duplications of Kunitz/BPTI genes prevailing in this genome. This also shows that the inference about tandem duplications of genes is highly dependent on the quality of the genome assembly and the lengths of genomic contigs. However, the short lengths of genomic contigs of Viperidae snakes prevent an in-depth insight into the real extent of tandem duplication events of the Kunitz/BPTI genes. As evident from the phylogenetic tree (Fig. 2), there were numerous duplication events in three Viperidae species with genomic data, and at least a few of them were in tandem.

### Evolutionary relationships in snake Kunitz/BPTI multigene families

A ML phylogeny of the snake Kunitz/BPTI multigene families (MF) has been made, with the inferred best fit model of amino acid sequence evolution for these proteins, which was JTT+G (Fig. 2). In the phylogenetic analysis we included Kunitz/BPTI proteins from venomous snakes only, since we focused on the Kunitz/BPTI genes which were functionally diversified (as evident from the presence of MF for these genes). From the snake Kunitz/BPTI ML tree we can infer how old the genes are and how and where they originated. A number of species-specific paralogs can be recognized in the ML tree, such as in *Daboia, Bungarus* etc. These paralogs group together in a species-specific manner, indicating that they are quite young gene duplicates. We also found older genes, where paralogs show dispersed pattern of distribution and are present in diverse species that belong to different snake families (Viperidae, Elapidae and even Colubridae). By searching with *V. ammodytes* trypsin inhibitor we found highly conserved genes that show up to 80% amino acid identity in Elapidae and Colubridae, which indicates that they belong to older Kunitz/BPTI genes that are about 50 My old^30^. These genes are indeed KU-WAP genes. ML phylogeny of venomous snake Kunitz/BPTI multigene families has shown that the observed diversity of these proteins is the consequence of several rounds of gene duplications at different time points. It is very likely that the observed diversity of venomous snake Kunitz/BPTI multigene families was strongly influenced by the predator/prey arms race.

### Strong positive selection is acting on the snake Kunitz/BPTI family

Evolution of snake Kunitz/BPTI genes was first evaluated under several standard models of sequence evolution as implemented in the Selecton. We used the site-specific model that accounts for the rate variation across the sites (Suppl. Table 2). Estimates using the BEB approach implemented in M8 model suggested that in Elapidae Kunitz/BPTIs up to 53% of the amino acid residues (codons) are under positive selection. In Viperidae snakes we found 28%, and in all venomous snakes 34% of codons under positive selection. Under the M8 model positive selection was evident in 28 sites of Elapidae Kunitz/BPTIs–each of these sites had a posterior expectation of ω higher than 1 with a posterior probability (PP) of at least 0.95. 16 sites have PP ≥ 0.95 and 12 sites have PP ≥ 0.99. In Viperidae Kunitz/BPTIs we found 15 positively selected sites, 7 with PP ≥ 0.95 and 8 sites with PP ≥ 0.99. In all venomous snake Kunitz/BPTIs M8 model detected 18 positively selected sites, only two sites with PP ≥ 0.95 and 16 sites with PP ≥ 0.99.

The MEC model^31^, which allows for positive selection, was compared with the M8a null model, which does not allow for positive selection. Comparison of the AICc scores (M8a: 10155; MEC: 10099) revealed that the MEC model fits the snake Kunitz/BPTI data better than the neutral M8a model. MEC model shows smaller amount of positively selected sites, 38% in Elapidae, 23% in Viperidae and 26% in all snake venom Kunitz/BPTIs. MEC model differs from the M models in that it allows instantaneous substitution between pairs of codons that differ at 2 or 3 codon positions and in its ability to take into account the different replacement probabilities between amino acids^31^. In Elapidae Kunitz/BPTIs MEC model detected 20 positively selected sites, 9 with PP ≥ 0.95 and 11 sites with PP ≥ 0.99. In Viperidae Kunitz/BPTIs MEC model detected 12 positively selected sites, 5 sites with PP ≥ 0.95 and 7 sites with PP ≥ 0.99. In all venomous snake Kunitz/BPTIs MEC model detected 14 positively selected sites, 4 sites with PP ≥ 0.95 and 10 sites with PP ≥ 0.99.

**Figure 3:**
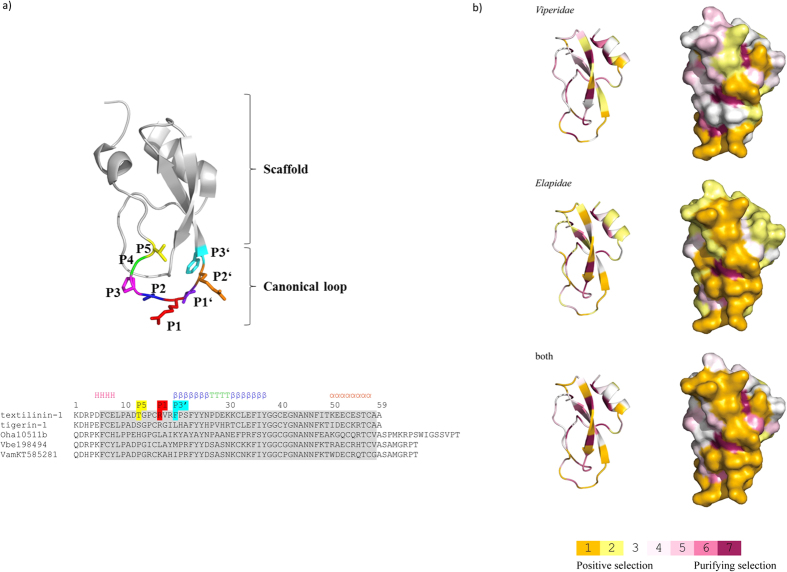
Positively selected amino acids on a 3D model. (**a)** Structure of textilinin-1 (3BYB) showing the canonical–antiprotease loop and the scaffold of the protein. Amino acid alignment of the selected snake Kunitz/BPTI proteins has highlighted P5 amino acid in yellow, P1 in red and P3′ in cyan as marked in the structure. Amino acids encoded in the second exon of Kunitz/BPTI genes were used for positive selection analyses (marked in grey). Secondary structure is represented with H–helix, β–β strand, T–turn, α–α helix. (**b)** Amino acids of the second exon in the structure of the textilinin-1 (3BYB) are represented as ribbons and surfaces, coloured in orange (with a posterior probability of 0.99) and yellow (with a posterior probability of at least 0.95) for positively selected sites and in magenta for purifying selection.

Visualization of site-specific ω estimations under the M8 and MEC models was obtained by translating the scores to a discrete color scale and their projection onto the 3D structure of textilinin-1^11^ (PDB: 3BYB) using the PyMol program^32^ (Fig. 3).

Positive selection, as detected by the Selecton, has been conclusively supported by the Single Likelihood Ancestral Counting (SLAC)^33^, Fixed Effects Likelihood (FEL), Fast Unconstrained Bayesian AppRoximation (FUBAR)^34^, Mixed Effects Model of Evolution (MEME)^35^ and the integrative selection analyses, implemented through the Datamonkey server^29^. MEME, SLAC, FEL, FUBAR and integrative approach have identified a very similar set of positively selected codons (Suppl. Table 3). The MEME model found the largest number of positively selected codons, followed by the SLAC, FUBAR, and FEL (Suppl. Table 3). Some sites were identified only by the Selecton models or by the Datamonkey models (Suppl. Tables 2 and 3). Evidence provided by various analyses (M8, MEC, SLAC, FEL, MEME, FUBAR and integrative selection analyses) revealed the strong influence of positive diversifying selection on the snake venom Kunitz/BPTI genes.

We compared the amount of positively selected sites in snake Kunitz/BPTI inhibitors with the only two previous studies that also included analysis of positively selected sites in Kunitz/BPTIs, one in vampire bats^25^ and the other in ticks^24^. No positively selected sites were found in tick Kunitz/BPTI inhibitors; however, they were found in tick channel inhibitors that evolved from the Kunitz/BPTI inhibitors^24^. The number of positively selected sites under M8 model in two-domain Kunitz/BPTIs of vampire bat is indeed much smaller than in snake venom Kunitz/BPTI proteins. In vampire bat Kunitz/BPTIs 4 positively selected sites were found under the M8 model in the first Kunitz domain and 5 in the second Kunitz domain. From our analysis it is evident that snake venom Kunitz/BPTI inhibitors possess the highest number of positively selected sites in the Kunitz/BPTI family. Our results clearly confirm that positive selection in snake venom Kunitz/BPTIs is much more influential than in vampire bat or ticks both in terms of the number of amino acids under selection and in the strength of selection.

### Positively selected amino acids in snake Kunitz/BPTI genes are located in functionally important regions

As a means to investigate selection patterns and to assess their influence on the structure and function of these proteins, we mapped the sites under selection on the 3D structure of snake Kunitz/BPTI protein textilinin-1^11^ (PDB: 3BYB). The numbering of amino acid positions is the same as in the textilinin-1. The locations of major structural elements in the textilinin-1 are the following: 3_10_ alpha-helix (positions from 5 to 8), canonical loop (positions from 15 to 20), beta sheet-1 (positions from 20 to 26), hairpin bend (positions from 27 to 29), beta sheet-2 (positions from 30 to 37), secondary binding loop (positions from 36 to 41) and alpha-helix (positions from 50 to 57)^11^.

In order to obtain some insights into the roles that positive selection might play, we mapped positively selected amino acids onto the 3D structure of the textilinin-1. We have mapped 18 positively selected sites detected under M8 model in all snake Kunitz/BPTI data set and found that five amino acids are located in the canonical loop (positions 15, 17, 18, 19 and 20), two are in the beta sheet-1 (positions 20 and 21), three in the hairpin bend (positions 27, 28 and 29), four in the beta sheet-2 (positions 31, 33, 34 and 36), two in the secondary binding loop (positions 36 and 41), two in the alpha-helix (positions 50 and 55), while positively selected amino acids 13 and 48 are not located in structurally important regions. Under the MEC model in the same data set we found 14 positively selected sites. Four amino acids are located in the canonical loop (positions 15, 17, 19 and 20), one in the beta sheet-1 (position 20), one in the hairpin bend (position 27), three in the beta sheet-2 (positions 31, 34 and 36), two in the secondary binding loop (positions 36 and 41), three in the alpha-helix (positions 50, 51 and 55) while positively selected amino acids 12 and 13 are not located in structurally important regions.

In Viperidae Kunitz/BPTIs we found 15 positively selected sites under the M8 model. Four amino acids are located in the canonical loop (positions 15, 17, 19 and 20), three in the beta sheet-1 (positions 20, 21 and 22), one in the hairpin bend (position 27), three in the beta sheet-2 (positions 31, 33 and 34), one in the secondary binding loop (position 41), three in the alpha-helix (positions 50, 52 and 55), while positively selected amino acid 48 is not located in the structurally important region. Under the MEC model we discovered 12 positively selected sites in the same data set. Three amino acids are located in the canonical loop (positions 17, 19 and 20), one in the beta sheet-1 (position 20), one in the hairpin bend (position 27), one in the beta sheet-2 (position 34), one in the secondary binding loop (position 41), four in the alpha-helix (positions 50, 52, 54 and 55), while positively selected amino acids 14 and 48 are not located in the structurally important regions.

In Elapidae Kunitz/BPTIs we discovered 28 positively selected sites under the M8 model. Only 11 sites were found to be negatively selected, which is a strong indication that Kunitz/BPTI proteins in Elapidae snakes are evolving under strong positive selection. Five positively selected amino acids are located in the canonical loop (positions 15, 17, 18, 19 and 20), four in the beta sheet-1 (positions 20, 21, 24 and 26), three in the hairpin bend (positions 27, 28 and 29), five in the beta sheet-2 (positions 30, 31, 33, 34 and 36), two in the secondary binding loop (positions 36 and 41), four in the alpha-helix (positions 50, 51, 54 and 55), one in the N-terminal helix (position 8), while four positively selected amino acids (positions 11, 12, 13 and 58) are not located in the structurally important region. Under the MEC model we discovered 20 positively selected sites in the same data set, five amino acids are located in the canonical loop (positions 15, 17, 18, 19 and 20), two in the beta sheet-1 (positions 20 and 21), two in the hairpin bend (positions 28 and 29), five in the beta sheet-2 (positions 30, 31, 33, 34 and 36), two in the secondary binding loop (positions 36 and 41), three in the alpha-helix (positions 50, 51 and 55), while three positively selected amino acids (positions 11, 12 and 13) are not located in the structurally important region.

Mapping of the positively selected codons that have been identified by MEME^35^, SLAC^33^, FEL, FUBAR^34^ and integrative approach has shown that they are mainly located in the same locations as in the M8 and MEC models (Suppl. Tables 2 and 3). For example, in the MEME model one positively selected site is located in the N-terminal helix (position 8), four are located in the canonical loop (positions 15, 17, 18 and 19), two in the beta sheet-1 (positions 21 and 22), three in the hairpin bend (positions 27, 28 and 29), two in the beta sheet-2 (positions 33 and 36), one in the secondary binding loop (position 36), three in the alpha-helix (positions 50, 51 and 55), while five positively selected sites (positions 11, 14, 44, 48 and 58) are not located in the structurally important region.

Although the Selecton and Datamonkey models slightly differ in the numbers of inferred positively selected sites, the majority of them are located in structurally and functionally important regions of the Kunitz/BPTI proteins, especially in the most important region: the canonical (antiproteinase) loop. In contrast to the analyses of positively selected sites in Kunitz/BPTIs of ticks^24^ and vampire bat^25^, where none or only few positively selected sites have been found, our analysis of venomous snake Kunitz/BPTIs shows strong and widespread positive selection that acts on structurally and functionally important regions of these proteins.

### The role of electrostatics in the function and evolution of snake venom Kunitz/BPTIs

Protein surface properties, such as electrostatic potential, play important roles in specific protein-protein and protein-ligand recognition^36^. Proteins and protein surfaces that interact with one another have electrostatic complementarity, and this property of protein-protein interaction is important for defining specificity. Intermolecular interactions involve associations between surfaces with complementary electrostatic potential. All protein charges, and not just the salt bridges or surface charges, contribute towards the value of electrostatic complementarity at the interface^37^.

The distribution of positively selected sites on the 3D Kunitz/BPTI structure has shown an interesting pattern. We demonstrated the presence of positively selected sites in the inhibitory loops 1 and 2, which form a binding interface. These loops are the most important parts of the Kunitz/BPTI proteins for the inhibition of the S1 serine peptidases^6^,^16^. The remainder of the Kunitz/BPTI structure forms the inhibitory scaffold, which is important in maintaining the loop conformation^3^,^16^. Surprisingly, we found unexpectedly high numbers of positively selected sites in the scaffold (Suppl. Table 4). Under all models, the positively selected sites in the scaffold strongly outnumber the positively selected sites in the inhibitory loops.

What is the consequence of the positively selected sites in the Kunitz/BPTI scaffold? When we investigated the alterations of charge and polarity for the amino acids identified as being under positive selection, the majority of amino acid sites exhibited at least one charged state alteration (Suppl. Fig. 3). Thus, the substitutions in positively selected sites could contribute with greater potency to the variations of the electrostatic potential on the surface of the snake venom Kunitz/BPTI proteins.

**Figure 4:**
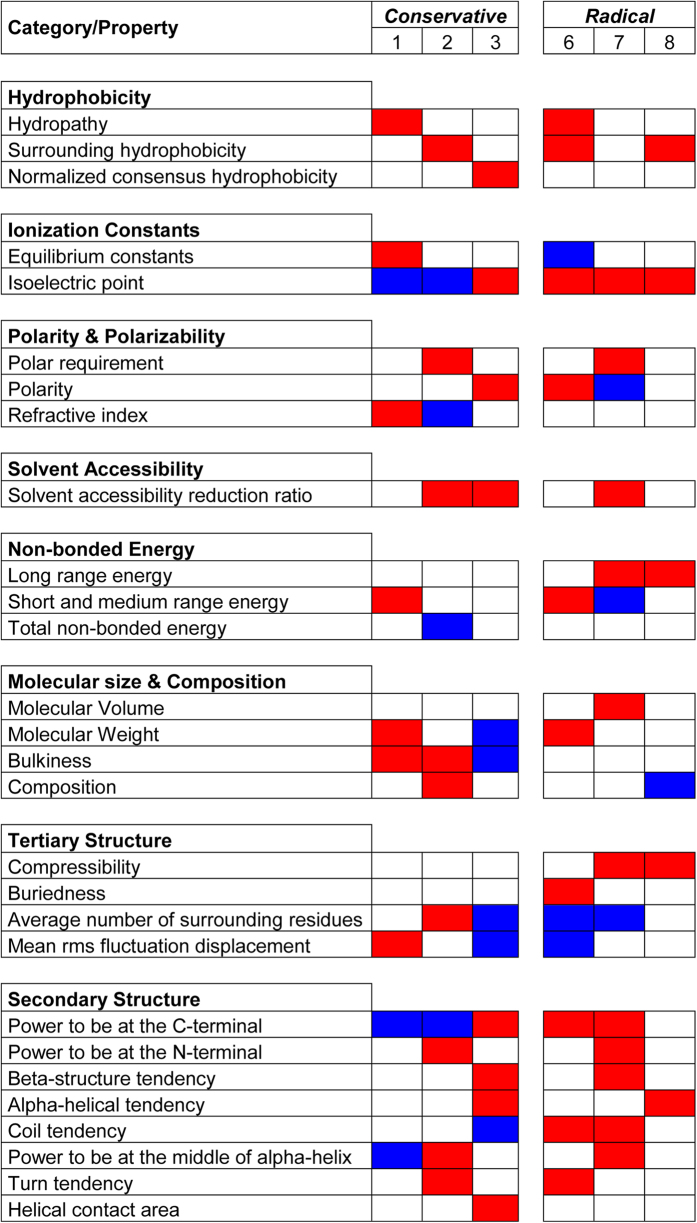
Amino acid properties under positive (red) and negative (blue) selection in snake venom Kunitz/BPTI genes. Conservative changes correspond to conservative categories 1 to 3 and radical changes to the categories 6 to 8 (*P* ≤ 0.001).

The program TreeSAAP v.3.2^38^ has been used to detect positive selection based on the physiochemical changes in protein sequences. The sliding window analysis in TreeSAAP identified 20 amino acid properties to be under positive destabilizing selection in the snake venom Kunitz/BPTI data set (P < 0.001) (Fig. 4; Suppl. Fig. 4). The following 8 amino acid properties are highly important for the electrostatics of the snake venom Kunitz/BPTI: hydropathy, surrounding hydrophobicity, isoelectric point, polar requirement, polarity, solvent accessible reduction ratio, long range energy as well as the short and medium range energy. These properties belong to the following categories: hydrophobicity, ionization constants, polarity and polarizability, solvent accessibility and non-bonded energy (Fig. 4; Suppl. Fig. 4). The remaing 12 amino acid properties belong to the following categories: molecular size and composition, secondary structure and tertiary structure (Fig. 4; Suppl. Fig. 4) and are not important for the electrostatics of the snake venom Kunitz/BPTI proteins.

**Figure 5:**
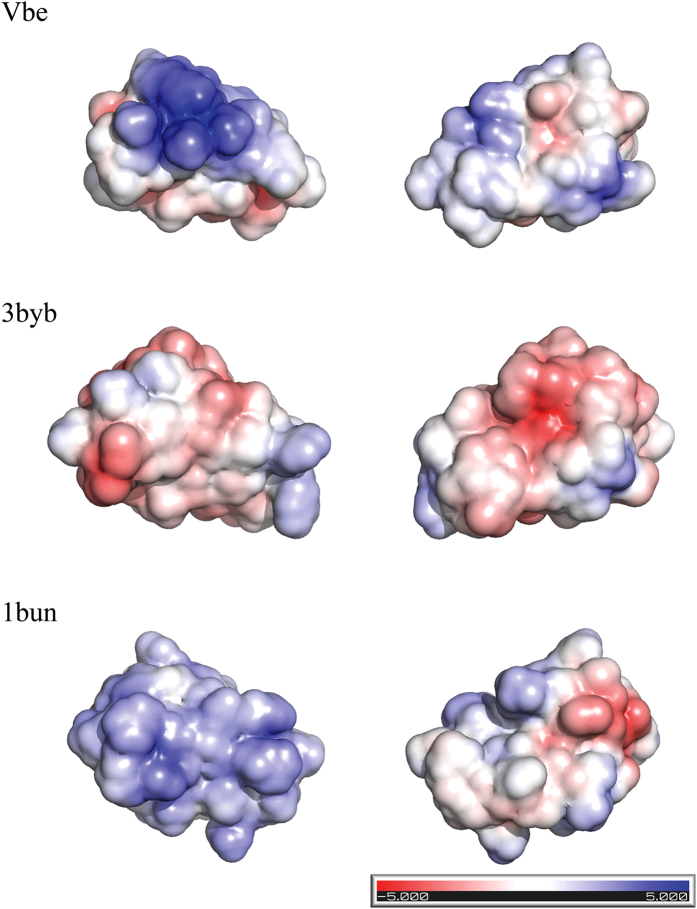
Electrostatic potentials of the *Vipera berus* Kunitz/BPTI inhibitor (VbeJTGP01198494), Textilinin-1 (3byb) and beta-bungarotoxin B2 chain (1bun). The electrostatic potential of the models was calculated by the Adaptive Poisson-Boltzmann Solver (APBS)^58^ using the PyMOL plug-in with the default parameter settings. ±5 kT/e electrostatic potential of the Vbe Kunitz/BPTI, Textilinin-1 and beta-bungarotoxin B2 chain was plotted on the solvent-accessible surface in PyMOL. The model of *Vipera berus* Kunitz/BPTI inhibitor (VbeJTGP01198494) was generated using Modeller^59^ with the structure PDB 3byb as a template. All images were prepared using The PyMOL Molecular Graphics System (Schrödinger, LLC).

The above analyses have demonstrated that the selection pressure has favored the acquisition of a distinct distribution of surface electrostatic potential in the snake venom Kunitz/BPTIs for more efficient inhibition of the diverse prey S1 serine peptidases. To better understand the potential functional changes during the evolution of snake venom Kunitz/BPTI multigene families, we analysed their 3D surface electrostatic potentials. Figure 5 shows the electrostatic surface potentials for a few snake venom Kunitz/BPTIs. We found that the distribution of surface electrostatic potential is markedly different among these proteins. The electrostatic potential of the surface might be affected more frequently with amino acid substitutions in the positively selected sites.

The BPTI fold is indeed quite rigid, without any dynamic conformational changes upon or before the inhibition of the target S1 peptidase^3^. Enzymes and their inhibitors have coevolved to form an interface with a high degree of surface complementarity^39^. Studies of the trypsin-BPTI complex have demonstrated the striking electrostatic and steric complementarity between the interacting surfaces in a “typical” protein-protein interaction. The driving forces for the binding of BPTI to trypsin are thus a complementary surface fit and electrostatic complementarity. The combination of the attractive and repulsive forces may help guide BPTI into the correct position for binding before actually contacting the active site^40^.

It is apparent that the consequence of the functional diversification in the snake venom Kunitz/BPTI multigene families has been the alteration of the electrostatic potential on the surface. The functional consequences can be associated with interactions and the recognition of the snake venom Kunitz/BPTIs with the diverse prey S1 serine peptidases.

### Why snakes need numerous and diverse Kunitz/BPTI inhibitors in their venoms?

The presence of diverse and numerous Kunitz/BPTI inhibitors in snake venoms (encoded by multigene families) represents an unusual situation^10^,^41^,^42^. It was demonstrated long ago that Kunitz/BPTI inhibitors are broad spectrum inhibitors of S1 family of serine peptidases^4^,^5^,^8^,^43^. In vertebrates S1 family peptidases belong to at least 8 putative clades (cell-mediated immunity, kallikrein, plasmin/HGF, degradative clades 1 to 3, fibrinolytic and ≫clotting and humoral immunity≪)^44^. In addition to trypsin and chymotrypsin (members of the degradative 1 and 2 clades), Kunitz/BPTI inhibitors can inhibit diverse representatives of serine peptidases from the majority of above mentioned clades^9^,^11^,^43^. The differences in the inhibition are attributable to the specificity and affinity of inhibition, and the inhibitory range can vary from micro to pico/nanomolar^45^.

S1 family of serine peptidases is the largest family of peptidases^46^ and can reach particularly large numbers in metazoans (from 100 to nearly 500) as well as in vertebrates (from 100 to over 300), where mammalian representatives − mouse (208), rat (217) and human (199)–possess quite large numbers of S1 peptidases (data obtained from the Merops Db). Since the vertebrate prey of venomous snakes possess very large numbers of S1 peptidases (from 100 to few hundreds), it is apparent why venomous snakes have large Kunitz/BPTI multigene families that can easily cope with the diverse S1 peptidases in their prey. It is interesting that the majority of venomous snakes also possess the combined KU and WAP domains, because both domains are inhibitors of S1 peptidases^47^. In this way the venomous snakes can easily increase their inhibitory range and the efficiency against the diverse prey S1 peptidases.

In snake venoms, Kunitz/BPTI inhibitors play important roles in inhibiting of quite large repertoires of prey S1 peptidases. Nevertheless, the snakes can also protect themselves in the case of attacks. In fact, the snake venom Kunitz/BPTI inhibitors and prey S1 peptidases are caught in a constant arms race–as is the case for other snake venom components^48^. Since venomous snakes attack diverse prey or use venom for defense, the cocktail of diversified Kunitz/BPTIs can serve as a good source of effective inhibitors of the S1 family of serine peptidases.

## Conclusions

Although we demonstrated adaptive evolution in snake venom Kunitz/BPTI proteins^10^, the exact mechanism of their evolution has remained unknown. In the present study we used a large collection of snake venom cDNAs and genes encoding Kunitz/BPTI family of proteins to analyse the action of site-specific positive selection and their impact on the structurally and functionally important parts of the Kunitz fold. By using diverse models we demonstrated the presence of large numbers of site-specific positively selected sites that can reach between 30–50% of the Kunitz domain. The mapping of the positively selected sites on the 3D model of Kunitz/BPTI inhibitors has shown that these sites are located in the structurally most important part of the molecule, in the inhibitory loops, but also in the Kunitz scaffold. Amino acid replacements are localized exclusively on the surface of the molecule, and the vast majority of replacements are causing the change of the charge. The consequence of these replacements is the change in the electrostatic potential on the surface of the Kunitz/BPTI proteins that may play an important role in the precise targeting of these inhibitors into the active site of S1 family of serine peptidases. The presence of the multigene families of Kunitz/BPTIs in venomous snakes can be explained by the target-oriented arms race, since the number of S1 peptidases in vertebrate preys can reach up hundreds of representatives. Since Kunitz/BPTIs are broad spectrum inhibitors, they can be functionally diversified to target numerous and diverse S1 peptidases in their prey. Comparison of the Kunitz/BPTI inhibitors from venomous snakes with ticks and vampire bats has demonstrated that they experienced strong and widespread action of site-specific positive selection only in the venomous snakes.

## Materials and Methods

### Screening the genomic library, cloning and sequencing of *V. ammodytes* Kunitz/BPTI genes

The genomic DNA library from *V. ammodytes*^49^, prepared in phage λGEM 12, was screened with a 155 bp long fragment of the *V. ammodytes* trypsin inhibitor. The preparation of ^35^S-labelled probe was described previously^10^. The library was screened by the plaque hybridization method^50^ under the following conditions: 24 h hybridization at 42 °C in hybridization buffer (6 X SSC (NaCl/Na-citrate), 50%(v/v) formamide, 5 X Denhardt’s solution, 0.5% (w/v) SDS and 500 μg denatured herring sperm DNA. The filters were washed in 4 X SSC/0.1% SDS for 15 min and 2 X SSC/0.1% SDS for 5 min at room temperature. Positive clones were purified by repeating the screening procedure. Phage DNA was isolated from plate lysates^50^ and digested with Eco RI, Hind III, Sac I and Bam HI restriction enzymes. Restriction fragments were separated by 0.7% agarose gel electrophoreses. Positive fragments were determined by Southern blotting using ^35^S-labelled probe described above and ligated into pUC19 vector. The inserts were sequenced on both strands with an ABI 310 sequence analyzer using BigDye chemistry (Applied Biosystems).

### PCR amplification of *V. ammodytes* Kunitz/BPTI genes

The sense, Kunitz5FLS (5′-RGAGAATAAATAGAGCSAGCAG-3′) and anti-sense, Kunitz3FLAS (5′-CACCWGARACCRAGARGGCAG-3′) oligonucleotide primers were based on highly conserved untranslated regions of *V. ammodytes* trypsin inhibitor^10^. PCR amplification of the additional *V. ammodytes* Kunitz/BPTI genes was performed in 100-μl volume with 1 μl of the *V. ammodytes* genomic library, each dNTP at 200 μM, 30 pmol of each primer, 1.5 mM MgCl_2_ and 2.5 units of AmpliTaq DNA polymerase. Optimization of the PCR led to the following two-step hot-start amplification program: initial denaturation 10 min at 94 °C, 30 cycles of 1 min at 94 °C denaturation and 1 min annealing at 48 °C and 2 min extension at 72 °C, and final extension at 72 °C for 7 min. The resulting PCR products were electrophoresed on 1% agarose gel and cloned into pGEM-T by pGEM-T Easy Vector System I kit (Promega). PCR amplified *V. ammodytes* Kunitz/BPTI genes were sequenced as described above.

### Database mining

The databases analyzed were the nonredundant, EST, TSA and WGS databases at the National Center for Biotechnology Information (NCBI) (http://www.ncbi.nlm.nih.gov). Comparisons were performed using the diverse BLAST tools^51^, with the E-value cutoff set to 10^−5^ and other parameters to default settings. Snake Kunitz/BPTI proteins, cDNAs or genes have been used as queries. DNA sequences were translated using the Translate program (http://web.expasy.org/translate). Orthologs and paralogs of the snake Kunitz/BPTI family have been identified in NCBI snake transcriptome, genome and proteome databases. The reference set of all representatives of the snake Kunitz/BPTI family is available in the Supplementary Table 5.

### Phylogenetic analysis of snake Kunitz/BPTI multigene families

We analysed 100 Kunitz/BPTI (including KU-WAP) genes and cDNAs, representing 32 venomous snake species (Suppl. Fig. 5). For the phylogenetic analysis we used 22 novel Kunitz/BPTI genes from four venomous snakes (*V. ammodytes, V. berus, C. mitchellii* and *O. hannah*). The Kunitz domain in the newly discovered representatives of the Kunitz/BPTI family has been identified using the SMART (http://smart.embl-heidelberg.de/), InterPro (https://www.ebi.ac.uk/interpro/) and Pfam (http://pfam.xfam.org/) domain databases. The protein sequences were aligned using Clustal Omega^52^. Molecular phylogenetic analyses of snake Kunitz/BPTI genes were conducted using the translated amino acid sequences. We used MEGA6^53^ to select best models of sequence evolution for the data from snake Kunitz/BPTI family. The best fit model of amino acid substitution for our data set was determined as JTT + G according to either BIC (Bayesian information criterion), AICc (Akaike information criterion) or lnL. Phylogenetic trees were reconstructed using the neighbor-joining (NJ) method^54^ and the maximum likelihood (ML) method^55^. The reliability of the resulting topologies was tested by the bootstrap method, 500 bootstrap replications were used. Condensed ML tree is shown as a Suppl. Fig. 6. As an outgroup, we used bovine BPTI. Phylogenetic analyses were performed with the program MEGA6^53^.

### Selection analyses

The Selecton server (Server for the Identification of Site-Specific Positive and Purifying Selection, version 2.4; http://selecton.tau.ac.il/index.html)^27^,^28^ has been used to identify the ratio of nonsynonymous and synonymous substitutions (dN/dS; termed ω) at each codon site based on an empirical Bayesian method. The pressure of selection can induce either purifying or positive selection at specific areas of the sequence where sites with ω values significantly higher or lower than one are an indication of positive or purifying selection, respectively^26^. 100 aligned coding sequences (exon 2) of the snake Kunitz/BPTI genes have been analysed. To identify codons under positive selection, we used two tests: M8 vs. M8a and MEC vs. M8a. M8 assumes a beta distribution, from 0 to 1, of ω for sites and an additional class of sites under positive selection (ω > 1), while M8a acts as a null model by fixing this last class of sites at ω = 1. Following these analyses, a likelihood ratio test was conducted on each model pair to determine if there were significant likelihood gains by allowing positive selection. Nested pairs of models (M8a and M8) and a non-nested pair (M8a and MEC) were compared using the likelihood ratio test implemented in the Selecton program. The results from Selecton version 2.4 are visualized with seven-color scale for representing the different types of selection. To identify the statistically significant levels of the results, the LRT was conducted to compare the nested models^56^. The Bayes empirical Bayes (BEB) approach^57^ was used to identify amino acids under positive selection by calculating the posterior probabilities that a particular amino acid belongs to a given selection class (neutral, conserved or highly variable). Sites with greater posterior probability (PP ≥ 95%) of belonging to the ‘ω > 1 class’ were inferred to be positively selected.

We further assessed the impact of positive selection using additional codon models to estimate the rates of synonymous and nonsynonymous substitutions^33^. Four methods implemented on the DATAMONKEY web server^29^ (http://www.datamonkey.org/), Single Likelihood Ancestral Counting (SLAC), Fixed Effects Likelihood (FEL), and FUBAR^34^, were used. After reconstructing ancestral sequences, SLAC compares normalized expected and observed numbers of synonymous and nonsynonymous substitutions per variable site. FEL compares the instantaneous synonymous site rate (α) and the instantaneous nonsynonymous site rate (β) on a per site basis, without assuming a prior dN/dS distribution. Sites with cut-off values of P < 0.1in SLAC and FEL and PP < 0.9 in FUBAR were considered as candidates to have evolved under positive selection. In all analyses performed in DATAMONKEY, the most suited model of evolution for each data set, directly estimated on this web server, was used. In addition, the mixed effects model of evolution (MEME), a branch-site method incorporated in the DATAMONKEY server, was used to test for both pervasive and episodic diversifying selection^35^. MEME is a generalization of FEL but models variable ω across lineages at individual sites, restricting ω to be ≤1 in a proportion p of branches and unrestricted at a proportion (1 − p) of branches per site. Positive selection was inferred with this method for a P value < 0.05.

PyMOL^32^ was used to visualize the structure and locations of the positively selected sites that have been identified by the likelihood ratio tests as well as for the visualization of the electrostatic potential maps.

### Investigating selective influences

Amino acid substitutions have a wide range of effects on a protein depending on the difference in physicochemical properties and location in the protein structure. This approach provides further resolution to differentiating between types of selective pressures with the ability to detect positive and negative and stabilizing and destabilizing selection and offers insights into the structural and functional consequences of the identified residues under selection. Significant physicochemical amino acid changes among residues in snake Kunitz/BPTI genes were identified by the algorithm implemented in TreeSAAP^38^, which compares the observed distribution of physicochemical changes inferred from a phylogenetic tree with an expected distribution based on the assumption of completely random amino acid replacement expected under the condition of selective neutrality. The evaluation of the magnitude of property change at nonsynonymous residues and their location on a protein 3D structure may provide important insight into the structural and functional consequences of the substitutions. Eight magnitude categories (1 to 8) represent one-step nucleotide changes in a codon and rank the correspondent variation in a property scale of the coded amino acid. Categories 1 to 3 indicate small variation in the amino acid characteristics while categories 6 to 8 represent the most radical substitutions. By accounting for the property changes across the data set, a set of relative frequencies changes for each category is obtained allowing to test the null hypothesis under the assumption of neutral conditions [65]. The categories for which the observed numbers of amino acid replacements in the data set is significantly different from the null model (z-scores > 1.645; P < 0.05) are considered as being potentially affected by selective pressures. Here we focus on amino acid differences that correspond to radical physicochemical variation (positive-destabilizing selection) and are expected to be linked with significant changes in function. TreeSAAP categorizes each amino acid site by positive and negatively destabilizing using 31 properties. To detect strong directional selective pressure, only changes corresponding to categories 6 to 8 (the most radical property change categories) at the P ≤ 0.001 level were considered. The total number of changes per site is the sum of those occurring in each branch of the phylogeny.

## Additional Information

**Accession codes**: All new sequences from this study were submitted to GeneBank database under accession numbers KT585273–KT585281.

**How to cite this article**: Župunski, V. and Kordiš, D. Strong and widespread action of site-specific positive selection in the snake venom Kunitz/BPTI protein family. *Sci. Rep.* **6**, 37054; doi: 10.1038/srep37054 (2016).

**Publisher's note:** Springer Nature remains neutral with regard to jurisdictional claims in published maps and institutional affiliations.

## Supplementary Material

Supplementary Information

## References

[b1] Rawlings N. D., Tolle D. P. & Barrett A. J. Evolutionary families of peptidase inhibitors. Biochem. J. 378, 705–716 (2004). 14705960 10.1042/BJ20031825 PMC1224039

[b2] Ascenzi P. . The bovine basic pancreatic trypsin inhibitor (Kunitz inhibitor): a milestone protein. Curr. Protein Pept. Sci. 4, 231–251 (2003). 12769721 10.2174/1389203033487180

[b3] Krowarsch D., Cierpicki T., Jelen F. & Otlewski J. Canonical protein inhibitors of serine proteases. Cell. Mol. Life Sci. 60, 2427–2444 (2003). 14625687 10.1007/s00018-003-3120-x PMC11138524

[b4] Bode W. & Huber R. Structural basis of the endoproteinase-protein inhibitor interaction. Biochim. Biophys. Acta 1477, 241–252 (2000). 10708861 10.1016/s0167-4838(99)00276-9

[b5] Czapinska H., Otlewski J., Krzywda S., Sheldrick G. M. & Jaskolski M. High-resolution structure of bovine pancreatic trypsin inhibitor with altered binding loop sequence. J. Mol. Biol. 295, 1237–1249 (2000). 10653700 10.1006/jmbi.1999.3445

[b6] Huber R. . Structure of the complex formed by bovine trypsin and bovine pancreatic trypsin inhibitor. Crystallographic refinement at 1.9 Å resolution. J. Mol. Biol. 89, 73–101 (1974). 4475115 10.1016/0022-2836(74)90163-6

[b7] Schechter I. & Berger A. On the size of the active site in proteases. Biochem. Biophys. Res. Commun. 27, 157–162 (1967). 6035483 10.1016/s0006-291x(67)80055-x

[b8] Laskowski M. Jr. & Kato I. Protein inhibitors of proteinases. Annu. Rev. Biochem. 49, 593–626 (1980). 6996568 10.1146/annurev.bi.49.070180.003113

[b9] Ritonja A., Turk V. & Gubenšek F. Serine proteinase inhibitors from *Vipera ammodytes* venom. Isolation and kinetic studies. Eur. J. Biochem. 133, 427–432 (1983). 6602050 10.1111/j.1432-1033.1983.tb07481.x

[b10] Župunski V., Kordiš D. & Gubenšek F. Adaptive evolution in the snake venom Kunitz/BPTI protein family. FEBS Lett. 547, 131–136 (2003). 12860400 10.1016/s0014-5793(03)00693-8

[b11] Millers E. K. . Crystal structure of textilinin-1, a Kunitz-type serine protease inhibitor from the venom of the Australian common brown snake (*Pseudonaja textilis*). FEBS J. 276, 3163–3175 (2009). 19490116 10.1111/j.1742-4658.2009.07034.x

[b12] Gilquin B. . Conformational and functional variability supported by the BPTI fold: solution structure of the Ca^2+^ channel blocker calcicludine. Proteins 34, 520–532 (1999). 10081964

[b13] Lancelin J. M., Foray M. F., Poncin M., Hollecker M. & Marion D. Proteinase inhibitor homologues as potassium channel blockers. Nat. Struct. Biol. 1, 246–250 (1994). 7544683 10.1038/nsb0494-246

[b14] Kwong P. D., McDonald N. Q., Sigler P. B. & Hendrickson W. A. Structure of β2-bungarotoxin: potassium channel binding by Kunitz modules and targeted phospholipase action. Structure 3, 1109–1119 (1995). 8590005 10.1016/s0969-2126(01)00246-5

[b15] Eng W. S. . Kunitz Peptides in Venomous Reptiles and Their Toxins: Evolution, Pathophysiology, and Biodiscovery (ed. Fry B. G.) 281–290 (Oxford University Press, 2015).

[b16] Bode W. & Huber R. Natural protein proteinase inhibitors and their interaction with proteinases. Eur. J. Biochem. 204, 433–451 (1992). 1541261 10.1111/j.1432-1033.1992.tb16654.x

[b17] Harvey A. L. & Robertson B. Dendrotoxins: structure-activity relationships and effects on potassium ion channels. Curr. Med. Chem. 11, 3065–3072 (2004). 15579000 10.2174/0929867043363820

[b18] Yang W. . BF9, the first functionally characterized snake toxin peptide with Kunitz-type protease and potassium channel inhibiting properties. J. Biochem. Mol. Toxicol. 28, 76–83 (2014). 24243656 10.1002/jbt.21538

[b19] Cheng Y. C., Yan F. J. & Chang L. S. Taiwan cobra chymotrypsin inhibitor: cloning, functional expression and gene organization. Biochim. Biophys. Acta 1747, 213–220 (2005). 15698956 10.1016/j.bbapap.2004.11.006

[b20] Cheng Y. C., Chen K. C., Lin S. K. & Chang L. S. Divergence of genes encoding B chains of beta-bungarotoxins. Toxicon 47, 322–329 (2006). 16457863 10.1016/j.toxicon.2005.11.009

[b21] Chang L. S., Wang J. J., Cheng Y. C. & Chou W. M. Genetic organization of *Bungarus multicinctus* protease inhibitor-like proteins. Toxicon 51, 1490–1495 (2008). 18471842 10.1016/j.toxicon.2008.03.025

[b22] St Pierre L. . Common evolution of waprin and kunitz-like toxin families in Australian venomous snakes. Cell. Mol. Life Sci. 65, 4039–4054 (2008). 18979207 10.1007/s00018-008-8573-5 PMC11131763

[b23] Doley R., Pahari S., Reza M. A., Mackessy S. P. & Kini R. M. The Gene structure and evolution of ku-wap-fusin (Kunitz waprin fusion protein), a novel evolutionary intermediate of the Kunitz serine protease inhibitors and waprins from Sistrurus catenatus (massasauga rattlesnake) venom glands. Open Evol. J. 4, 31–41 (2010).

[b24] Dai S. X., Zhang A. D. & Huang J. F. Evolution, expansion and expression of the Kunitz/BPTI gene family associated with long-term blood feeding in *Ixodes scapularis*. BMC Evol. Biol. 12, 4 (2012). 22244187 10.1186/1471-2148-12-4 PMC3273431

[b25] Low D. H. . Dracula’s children: molecular evolution of vampire bat venom. J. Proteomics 89, 95–111 (2013). 23748026 10.1016/j.jprot.2013.05.034

[b26] Yang Z. & Nielsen R. Codon-substitution models for detecting molecular adaptation at individual sites along specific lineages. Mol. Biol. Evol. 19, 908–917 (2002). 12032247 10.1093/oxfordjournals.molbev.a004148

[b27] Doron-Faigenboim A., Stern A., Mayrose I., Bacharach E. & Pupko T. Selecton: a server for detecting evolutionary forces at a single amino-acid site. Bioinformatics 21, 2101–2103 (2005). 15647294 10.1093/bioinformatics/bti259

[b28] Stern A. . Selecton 2007: advanced models for detecting positive and purifying selection using a Bayesian inference approach. Nucleic Acids Res. 35, W506–W511 (2007). 17586822 10.1093/nar/gkm382 PMC1933148

[b29] Delport W., Poon A. F., Frost S. D. & Kosakovsky Pond S. L. Datamonkey 2010: a suite of phylogenetic analysis tools for evolutionary biology. Bioinformatics 26, 2455–2457 (2010). 20671151 10.1093/bioinformatics/btq429 PMC2944195

[b30] Hsiang A. Y. . The origin of snakes: revealing the ecology, behavior, and evolutionary history of early snakes using genomics, phenomics, and the fossil record. BMC Evol. Biol. 15, 87 (2015). 25989795 10.1186/s12862-015-0358-5 PMC4438441

[b31] Doron-Faigenboim A. & Pupko T. A combined empirical and mechanistic codon model. Mol. Biol. Evol. 24, 388–397 (2007). 17110464 10.1093/molbev/msl175

[b32] DeLano W. L. The PyMOL Molecular Graphics System. DeLano Scientific, San Carlos, CA, USA (2002).

[b33] Kosakovsky Pond S. L., Frost S. D. W. & Pond S. L. K. Not so different after all: a comparison of methods for detecting amino acid sites under selection. Mol. Biol. Evol. 22, 1208–1222 (2005). 15703242 10.1093/molbev/msi105

[b34] Murrell B. . FUBAR: a fast, unconstrained bayesian approximation for inferring selection. Mol. Biol. Evol. 30, 1196–1205 (2013). 23420840 10.1093/molbev/mst030 PMC3670733

[b35] Murrell B. . Detecting individual sites subject to episodic diversifying selection. PLoS Genet. 8, e1002764 (2012). 22807683 10.1371/journal.pgen.1002764 PMC3395634

[b36] Honig B. & Nicholls A. Classical electrostatics in biology and chemistry. Science 268, 1144–1149 (1995). 7761829 10.1126/science.7761829

[b37] McCoy A. J., Chandana Epa V. & Colman P. M. Electrostatic complementarity at protein/protein interfaces. J. Mol. Biol. 268, 570–584 (1997). 9159491 10.1006/jmbi.1997.0987

[b38] Woolley S., Johnson J., Smith M. J., Crandall K. A. & McClellan D. A. TreeSAAP: selection on amino acid properties using phylogenetic trees. Bioinformatics 19, 671–672 (2003). 12651734 10.1093/bioinformatics/btg043

[b39] Gabb H. A., Jackson R. M. & Sternberg M. J. Modelling protein docking using shape complementarity, electrostatics and biochemical information. J. Mol. Biol. 272, 106–120 (1997). 9299341 10.1006/jmbi.1997.1203

[b40] Weiner P. K., Langridge R., Blaney J. M., Schaefer R. & Kollman P. A. Electrostatic potential molecular surfaces. Proc. Natl. Acad. Sci. USA 79, 3754–3758 (1982). 6285364 10.1073/pnas.79.12.3754 PMC346505

[b41] Cardle L. & Dufton M. J. Foci of amino acid residue conservation in the 3D structures of the Kunitz BPTI proteinase inhibitors: how do variants from snake venom differ? Protein Eng. 10, 131–136 (1997). 9089812 10.1093/protein/10.2.131

[b42] Pritchard L. & Dufton M. J. Evolutionary trace analysis of the Kunitz/BPTI family of proteins: functional divergence may have been based on conformational adjustment. J. Mol. Biol. 285, 1589–1607 (1999). 9917399 10.1006/jmbi.1998.2437

[b43] Fritz H. & Wunderer G. Biochemistry and applications of aprotinin, the kallikrein inhibitor from bovine organs. Arzneimittelforschung 33, 479–494 (1983). 6191764

[b44] Krem M. M., Rose T. & Di Cera E. Sequence determinants of function and evolution in serine proteases. Trends Cardiovasc. Med. 10, 171–176 (2000). 11239798 10.1016/s1050-1738(00)00068-2

[b45] Chen C. . Solution structure of a Kunitz-type chymotrypsin inhibitor isolated from the elapid snake *Bungarus fasciatus*. J. Biol. Chem. 276, 45079–45087 (2001). 11562364 10.1074/jbc.M106182200

[b46] Page M. J. & Di Cera E. Serine peptidases: classification, structure and function. Cell. Mol. Life Sci. 65, 1220–1236 (2008). 18259688 10.1007/s00018-008-7565-9 PMC11131664

[b47] Moreau T. . Multifaceted roles of human elafin and secretory leukocyte proteinase inhibitor (SLPI), two serine protease inhibitors of the chelonianin family. Biochimie 90, 284–295 (2008). 17964057 10.1016/j.biochi.2007.09.007

[b48] Kordiš D. & Gubenšek F. Adaptive evolution of animal toxin multigene families. Gene 261, 43–52 (2000). 11164036 10.1016/s0378-1119(00)00490-x

[b49] Kordiš D. & Gubenšek F. Ammodytoxin C gene helps to elucidate the irregular structure of Crotalinae group II phospholipase A_2_ genes. Eur. J. Biochem. 240, 83–90 (1996). 8797839 10.1111/j.1432-1033.1996.0083h.x

[b50] Sambrook J., Fritsch E. F. & Maniatis T. Molecular Cloning: A Laboratory Manual, 2nd edn., (Cold Spring Harbor Laboratory Press, 1989).

[b51] Gertz E. M., Yu Y. K., Agarwala R., Schäffer A. A. & Altschul S. F. Composition-based statistics and translated nucleotide searches: improving the TBLASTN module of BLAST. BMC Biol. 4, 41 (2006). 17156431 10.1186/1741-7007-4-41 PMC1779365

[b52] Sievers F. . Fast, scalable generation of high-quality protein multiple sequence alignments using Clustal Omega. Mol. Syst. Biol. 7, 539 (2011). 21988835 10.1038/msb.2011.75 PMC3261699

[b53] Tamura K., Stecher G., Peterson D., Filipski A. & Kumar S. MEGA6: Molecular Evolutionary Genetics Analysis version 6.0. Mol. Biol. Evol. 30, 2725–2729 (2013). 24132122 10.1093/molbev/mst197 PMC3840312

[b54] Saitou N. & Nei M. The neighbor-joining method: a new method for reconstructing phylogenetic trees. Mol. Biol. Evol. 4, 406–425 (1987). 3447015 10.1093/oxfordjournals.molbev.a040454

[b55] Guindon S. . New algorithms and methods to estimate maximum-likelihood phylogenies: assessing the performance of PhyML 3.0. Syst. Biol. 59, 307–321 (2010). 20525638 10.1093/sysbio/syq010

[b56] Anisimova M., Bielawski J. P. & Yang Z. Accuracy and power of the likelihood ratio test in detecting adaptive molecular evolution. Mol. Biol. Evol. 18, 1585–1592 (2001). 11470850 10.1093/oxfordjournals.molbev.a003945

[b57] Yang Z., Wong W. S. & Nielsen R. Bayes empirical Bayes inference of amino acid sites under positive selection. Mol. Biol. Evol. 22, 1107–1118 (2005). 15689528 10.1093/molbev/msi097

[b58] Baker N. A., Sept D., Joseph S., Holst M. J. & McCammon J. A. Electrostatics of nanosystems: application to microtubules and the ribosome. Proc. Natl. Acad. Sci. USA 98, 10037–10041 (2001). 11517324 10.1073/pnas.181342398 PMC56910

[b59] Eswar N. . Comparative protein structure modelling using MODELLER. Curr. Protoc. Protein Sci. 50 **(2.9**), 2.9.1–2.9.31 (2007). 10.1002/0471140864.ps0209s50 18429317

