# Supplementary material for: Strong and widespread action of site-specific positive selection in the snake venom Kunitz/BPTI protein family
Source: Sci Rep. 2016 Nov 14;6:37054. doi: 10.1038/srep37054 (PMC5107962; doi:10.1038/srep37054)
Supplement: Supplementary Information [file srep37054-s1.doc]

**Supplementary Information**

**Strong and widespread action of site-specific positive selection in the snake venom Kunitz/BPTI protein family**

Vera Župunski and Dušan Kordiš*^, 1^

Department of Chemistry and Biochemistry, Faculty of Chemistry and Chemical Technology, University of Ljubljana, Ljubljana, Slovenia.

*Department of Molecular and Biomedical Sciences, Josef Stefan Institute, Ljubljana, Slovenia.

^1^Corresponding author: Dušan Kordiš, [dusan.kordis@ijs.si](mailto:dusan.kordis@ijs.si)

*

KT585281 MSSGGLLLLLGLLTLWAELTPVSGQDHPKFCYLPADPGRCKAHIPRFYYDSASNKCNKFI

KT585276 MSSGGLLLLLGLLTLWAELTPISGRDRPKFCHLPADPGICKAHTPRFYYDSASNKCKQFS

KT585273 MSSGGLLLLLGLLTLWAELTPVSTRDRPKFCYLPADPGRCLAYIPSFYYDSASNKCKKFI

KT585278 MSSGGLLLLLGLLTLWAELTPVSTRDRPKFCYLPADPGRCLAYIPSFYYDSASNKCKKFI

KT585280 MSSGGLLLLLGLLTLWAELTPVSTRDRPKFCYLPADPGRCLAYIPSFYYDSASNKCKKFI

KT585274 MSSGGLLLLLGLLTLWAELTPVSTRDRPKFCYLPADPGRCLAEMPRFYYNPASNKCKEFI

KT585277 MSSGGLLLLLGLLTLWAELTPVSTRDRPKFCYLPADPGRCLAYMPRFYYNPASNKCKEFI

KT585275 MSSGGLLLLLGLLTLWAELSPVSTRDRPKFCYLPADPGRCLAYMPRFYYNPASNKCKEFI

KT585279 MSSGGLLLLLGLLTLWAELTPVSTRDRPKFCYLPADPGRCLAYMPRFYYNPASNKCKEFI

*******************:*:* :*:****:****** * * * ***: *****::*

KT585281 YGGCPGNANNFKTWDECRQTCGASAMGRPT-----

KT585276 FGGCPGNANNSKTWDECRHTCVASGKGIQ------

KT585273 YGGCRGNANNFKTWDECRHTCVAS--GIQPRIASN

KT585278 YGGCRGNANNFKTWDECRHTCVAS--GIQPRIASN

KT585280 YGGCRGNANNFKTWDECRHTCVAS--GIQPRIASN

KT585274 YGGCRGNANNFKTWDECRHTCVAS--GIQPRIASN

KT585277 YGGCRGNANNFKTWDECRHTCVAS--GIHPRIASN

KT585275 YGGCRGNANNFKTWDECRHTCVASGKGIQPRIASN

KT585279 YGGCRGNANNFKTWDECRHTCVASGKGIQPRIASN

:*** ***** *******:** ** *

**Suppl. Fig. 1. Amino acid alignment of the *Vipera ammodytes* Kunitz/BPTI inhibitors.** Signal peptide is marked in yellow, C-terminal extension in green, antiproteinase site is marked with a red asterisk (*).

**>KT585273, 2018 bp**

TTTCAGACCTTGTGTGTATGTGTGTCTGTGTGTATGTGTGTGTGTGTGTG

TGTGTATTTGCGGATTTGTGCCTTCGTTCTTGGACTTTGCAACCCTGGGA

CTAGACGGAACAATACTAGACTCAACAGAACTTCACATTTGGGAATGGCA

ATAGGGGCCTGGAGTGTGTGTGTGTGAGGGGCGGAGACTAGACGTGGCAT

CAGGGACCTGCCTTCCTTTCCCCAACCGTCCAGCCAATGCAGCTCAGAGG

ATTCCCCACCTCTTGTTCAACCAACTAATGAAAGAGGAAAGAAATGAAAT

CCTCACTCTATTACTGCATCCTGCTTCCTGGATGTGAAATTCCCAGTTGA

GAGAATAAATAGAGCGAGCAGCCCGGGTGTGGGGCAGACGGCTTCATC**AT**

**M**

**GTCTTCTGGAGGTCTTCTTCTCCTGCTGGGACTCCTCACCCTCTGGGCAG**

**S S G G L L L L L G L L T L W A**

**AGCTGACCCCCGTCTCCACCCGGGACCGTCCAA**GTGAGTTTCCAGAATGA

**E L T P V S T R D R P**

TCTTGAAACCCTTTGCTGGGAGGGGCTGACGTGATCAGAGGGCCGAATTC

TGCCCCAGGAAGGGAAACTCAAGAGACTCTGGTCTAGAACCTCTGGCTTG

GTGGTGCCCCTTTGGGAAGGTTCTTGAGATGGTGGTTGGGTGTCAGCTGA

ACAGGAGACAGAAATCGGAGGGGCCCCCAAGAAAGAGAAATGCAATTTCA

GGGGAAGGCTGACCTATCTTTACTCAATGCAACAGCAGAAACCGGAGTTT

AAGGCACAGGATCCATCATTCAGCCATCTGGTCAACCCAGGAAGCCAAAT

TAGAACATTCCAGATGGCTCTTGATCATGTGCAGACTGAGCATGCTAGGA

AAGCACAAGGTTGGGGAAGTGCCTTAAATACGTTTGTGCTAGCTAATCAA

ATAACACACACAAACACACCTTTCCTTTTCCTGCAG**AGTTTTGTTATCTC**

**E F C Y L**

**CCTGCTGATCCTGGAAGATGTTTGGCCTATATACCTAGTTTCTACTACGA**

**P A D P G R C L A Y I P S F Y Y D**

**TTCGGCATCAAACAAATGTAAAAAATTTATTTACGGTGGATGCCGTGGGA**

**S A S N K C K K F I Y G G C R G**

**ATGCCAACAATTTTAAGACCTGGGATGAATGTCGCCACACCTGTGTTG**GT

**N A N N F K T W D E C R H T C V**

AAGTAGGGAAGGAGCTGAGGGCTGACTGCTTCATTGAACTGGGTGGCTTA

ATGTTATATCCTGGTTCTCACAACAAGCTATGATTGATTCAGTAGAAGGG

AAGGGAATTTTCAGGGCTGAACTGTGGAGTCCATTCTCCAAAGCACCTGA

GGGCAGGACAAGAAGCGACAGATGGAAACTCACCAAGGAGAGAAGCAACC

TGGAACTAGGAAGAAATTTCCTGGCAGTGAGAACAATTAATTAGTGGAAC

AGAAGCTGCCTCCAGAAATTGAGGGTGCTCCGTCACTGGAGGTTTTCAAG

AAGAGACTGGACAGCCACTTGTCTGGAATGGTGTAGGGTTTCCTGCCTAA

CCAGGGGGTTGGACTAGAAGACCTCCAAGGTGCCTTCCAACTCTGTTATT

CTATATTGCATGTTCTGCTTGAAACCCCCAGTGATGGAGCCCCATAACTC

CAAGGGGGCAGCTACCCCATTGCTTCCTGGGTCTCCCAATCAGGTCATTC

CTCCAGATTTCAATTCGAGTCTCTGTGGTTGTACGATTAGCCAGGATGAA

GCACCGCCTGCAGTCCTGGGCAGAAATCTCATGTCTGAATGTCTCTTTTT

ACAG**CATCCGGAATACAACCCCGAATTGCGTCTAATTAGCCAACCTCACT**

**A S G I Q P R I A S N ***

**GAGAGGCTTTCCTTCCATCCTGGATTATTCTGGAGACCCTCCCCCCAAAC**

**CCAACCTGGCTTCATCCCTTCTATTCTGCAATAAAGCTTTGTTTCTCGCC**

**TG**CCTTCTTGGTCTCAGGTGCCTCTTTCTGCCCCCTTGAGGGTTTTCCCT

CCCCACAAAAGCCCCAGTGTGGCTTTCAGAGGGAATCCACAGGGGAGACA

CACCTCGGAGACAGAAACTCTTAAAAAACACCAGCTGCCTGCAATTAGTG

CAGGTTCGAGTCCACAGT

**>KT585281, 1452 bp**

GCTTCATC**ATGTCTTCTGGAGGTCTTCTTCTCCTGCTGGGACTCCTCACC**

**M S S G G L L L L L G L L T**

**CTCTGGGCAGAGCTGACCCCCGTCTCCGGGCAGGACCATCCAA**GTGAGTT

**L W A E L T P V S G Q D H P**

TCCAGAATGATCTTGAAACCCTTTGCTGGGAGGGGCTGACGTGATCAGAG

GGCAGAGTTCTCCCCCACGGAGAGGAAACCAAGAACCCCAATCTAGAACC

TCTGGCTTGGTGGTGCCTCTTCTTGTAAGGTTCTTGAGATGGTGGTTGGG

TGTCAGCTGGAGACAGAAATCAGAGGGGCCCCCAAAAAAGAGAATTGTAT

TTTCAGGGGAAGGCTGACTTATTTTTACTCAATGCAACAGCAGAATCCGG

AGTTTAAGGAACAGGATCCATCATTCAGCCATCTGGTCCAACCCAGGAAG

CCAAATTAAAACATCCCAGATGGCTCTTGATCATGTGCAGACTGAGCATG

CTAGGAAAGCACAAGGTTGGGGAAGTGCCTTAGAGACGTTTCTGCTACCT

AATCAAATAACACACACAAACACACCTTTTTTTTTCCTACAG**AGTTTTGT**

**K F C**

**TATCTCCCTGCTGATCCTGGAAGATGTAAAGCCCATATCCCTCGTTTCTA**

**Y L P A D P G R C K A H I P R F Y**

**CTACGATTCGGCATCAAACAAATGTAACAAATTTATTTACGGTGGATGCC**

**Y D S A S N K C N K F I Y G G C**

**CTGGGAATGCCAACAATTTTAAGACCTGGGATGAATGTCGCCAAACCTGT**

**P G N A N N F K T W D E C R Q T C**

**GGTG**GTAAGTAGGGAAGGAGCTGAGGGCTGACTGCTTGATTGAACTGGGT

**G**

GGCTTAATGTTATATCCTGGTTCTCACAACAGGCTATGATGGATTCAGTA

GAAGGGAAGGGAATTTTCAGGGTTGAACTGTGGAGTCCATTCTCCAATTC

ACCTGAGGGCAGGACAAGAAGCGACAGATGGAAACTCATCAAGAAGAGAA

GCAACCTGGAAGTAGGAAGAAATTTCCTGGCAGTGAGAACAATTAATTAG

TGGAACAGAAGCTGCCTCCAGAAATTGAGGGTGCTTCGTCACTGGAGGTT

CTCAAGAAGAGACTGGACAGCCACTTGTCTGGAATGGTGTAGGGTTTCCT

GCCTAACCAGGGGGTTGGACTAGAAGACCTCCAAAGTCCCTTCCAATTCT

GTTATTCTATATTGCGTGTTCTGCTTGAAACCCCCAGTGATGGAGCCCCA

TAACTCCAGGGCGGCAGCTACCCCAGTGCTTCCGGGCTCTCCCAATCAGG

CCATTCCTCCAGATTTCAATTCGAGTCTCTGTGGTTGTACGATTAGCCAG

GATGAAGCACTGCCTGCAGTTCCTGGGCAGAAATCTCATGTCTGAATGTC

TCTTTTTACAG**CATCCGCAATGGGGAGACCCACCTGAATTGGGTCTAATT**

**A S A M G R P T ***

**TGCCAACTTCACTGAGAGGCTTTCCTTCCGTCCTGGATTATTCTGGAGAC**

**CCTCCCCCCAAACCCACCCTGGCTTCATCCCTTCTGCTCTACAATAAAGC**

**T**

**Suppl. Fig. 2. *Vipera ammodytes* chymotrypsin inhibitor (KT585273) and trypsin inhibitor (KT585281) genes.**


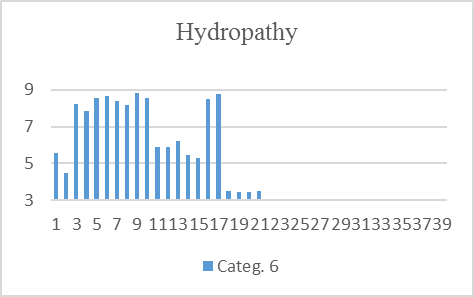

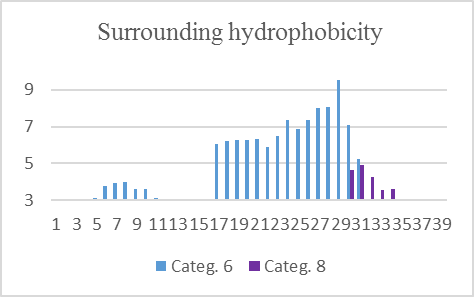


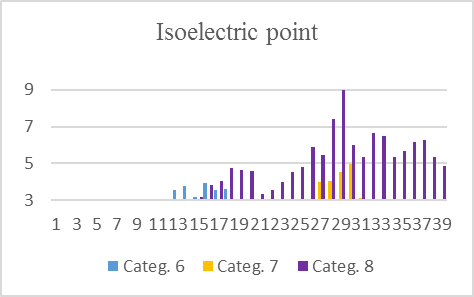

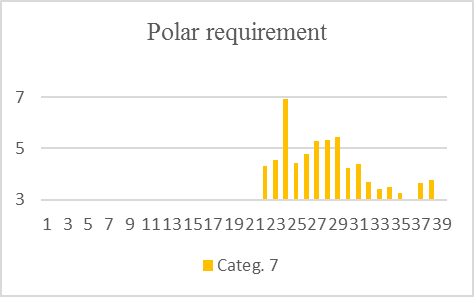


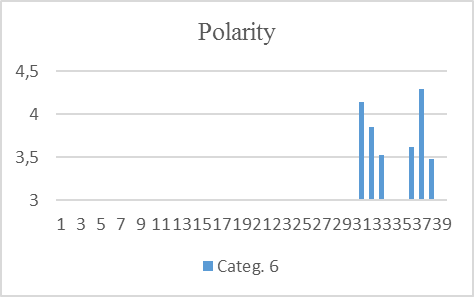

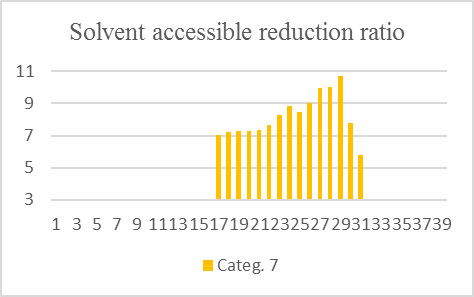


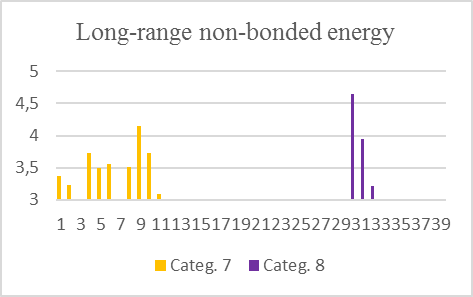

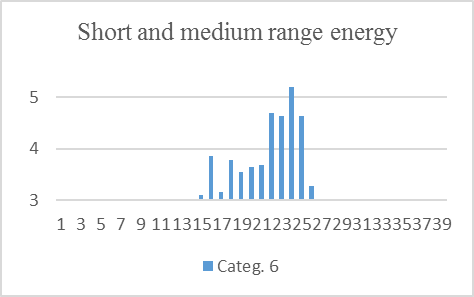


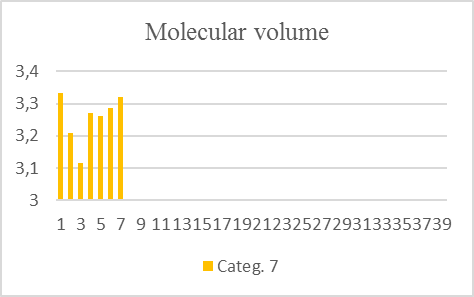

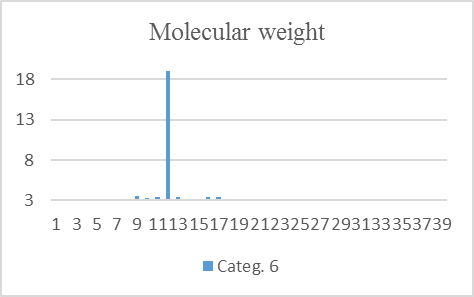


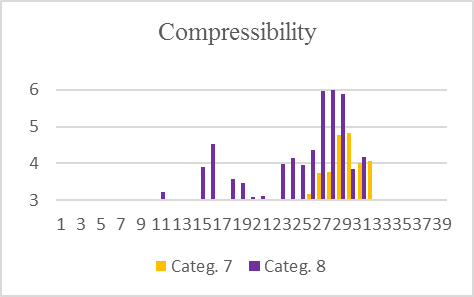

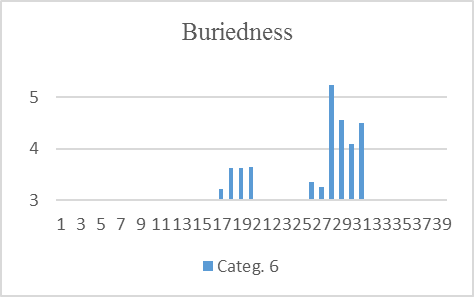


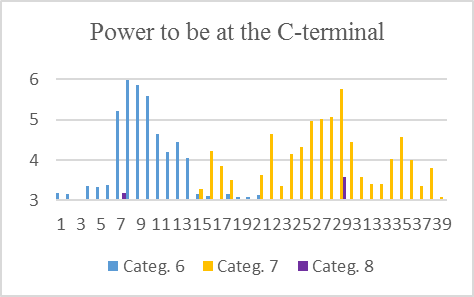

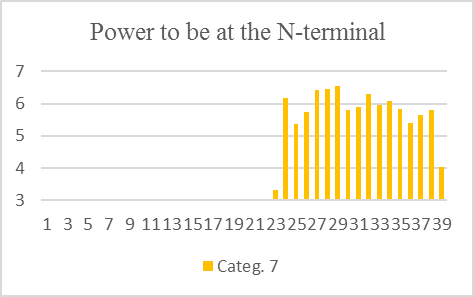


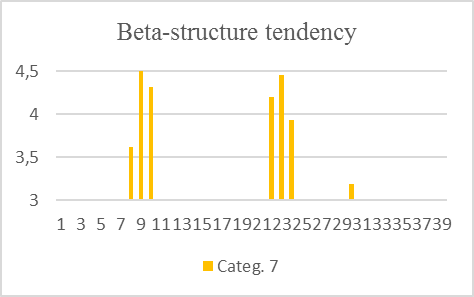

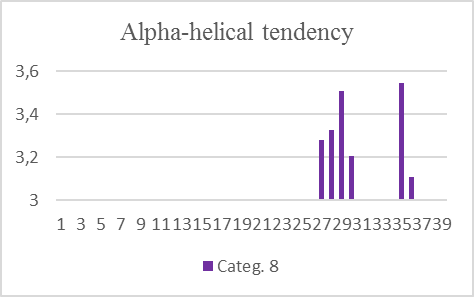


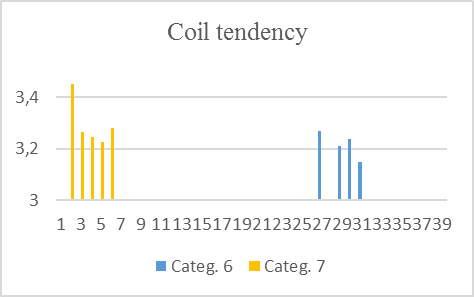

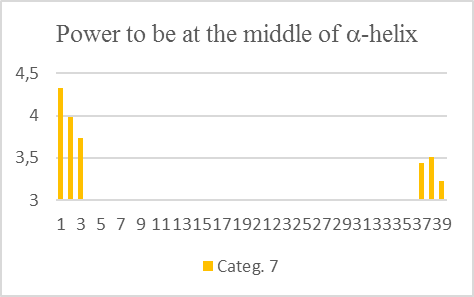


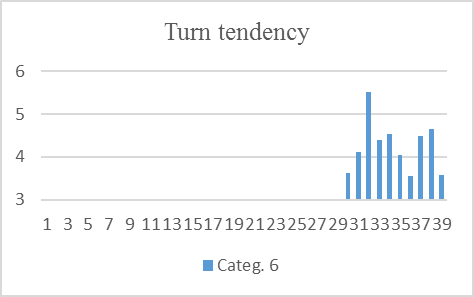


**Suppl. Fig. 4. The sliding window analysis of the amino acid properties under positive destabilizing selection in the snake venom Kunitz/BPTI genes.** Radical changes correspond to the categories 6 to 8 (*P* ≤0.001). A sliding window of 15 amino acids provided the best signal-to-noise ratio.

BmuAM939781 SCDKAPDTERCKRNVYAFYYNPSARDCLQFVYGGCDGNGKHFRSKALCLFHCH

BflAB112358 DCDKPPNKKRCTGHIPAFYYNPQRKTCERFSYGGCKGNGNHFKTPQLCMCHCH

BmuAJ242991 DCDKPPDKGNCGPVRRAFYYDTRLKTCKAFQYRGCNGNGNHFKSDHLCRCECL

BmuY12100 DCDKPPDKGNCGPVRRAFYYDTRLKTCKAFQYRGCNGNGNHFKTETLCRCECL

BcaAY057883 DCDKPPDKGNCGSVRRAFYYDTRLKTCKAFPYRGCNGNGNHFKTETLCRCECL

BmuY12101 DCDKPPDTKICQTVVRAFYYKPSAKRCVQFRYGGCNGNGNHFKSDHLCRCECL

BcaAB158300 DCDKPPDTRICQTVVRAFYYKPSEKRCVQFRYGGCKGNGNHFKSDHLCRCECL

BosAAD13685 FCLEPPYTGPCKARIIRYFYNAKAGLCQTFVYGGCRAKRNNFKSAEDCMRTCG

BcaAY057887 FCNVPPEPGRCNANVRAFYYNPRLRKCIEFTYGGCGGNANNFKSGGECKRACG

BmuAM939783 FCNVPPEPGRCNANVRAFYYNPRLRKCIEFSYGGCGGNANNFKSRGECKRTCA

CmiJPMF01217719 LCYLPPVHGPCKGHFHHFHYHSASNGCREFIYGGCQGNANNFKTRDECLYTCV

EcoGBUG01000066 LCYFPADPKICKVHVPHFYYDLASNQCKQFKFGECPSNANNFKTRDECHYTCV

BgaAY442289a FCNLPADLGPCKNYTGRFYYDSASNKCEVFIYGGCPGNANNFKTREECRKTCV

BgaAY442289b ICILPAELGPCDEYTGRFYYDSASNKCEVFIYGGCQGNANNFKTRDECRKTCV

DsiAM411368 FCYLPADPGECLAHMRSFYYDSESKKCKEFIYGGCHGNANKFPSRDKCRQTCG

DruDQ365980 FCYLPADPGECMAYIRSFHYDSESKKCKEFIYGGCHGNANNFPTRDKCRQTCR

BgaAY430402 FCNLPADTGPCKAYEPRFYYDSVSKECQKFTYGGCKGNSNNFESMDECRKTCV

BgaAY430413 FCYLPADTGPCMANFPRFYYDSASKKCKKFTYGGCHGNANNFETREECRKKCF

VamKT585281 FCYLPADPGRCKAHIPRFYYDSASNKCNKFIYGGCPGNANNFKTWDECRQTCG

VbeJTGP01201332 FCYLPADPGRCKAHIPRFYYDSASNKCKKFIYGGCRGNANNFKTWDECRQTCG

MleHE800183 FCYLPADPAECNAYMPRFYYDSASNKCKEFIYGGCRGNANNFKNRAECRHTCV

VbeJTGP01201339 FCYLPAEPGECNAYMPSFYYDSASNKCKKFIYGGCRGNANNFKTRDECHHTCV

VbeJTGP01198494 FCYLPADPGICLAYMPRFYYDSASNKCKKFIYGGCGGNANNFKTRAECRHTCV

VamKT585273 FCYLPADPGRCLAYIPSFYYDSASNKCKKFIYGGCRGNANNFKTWDECRHTCV

VamAY217782 FCYLPADPGRCLAYMPSFYYDSASNKCKKFIYGGCRGNANNFKTWDECRHTCV

VamKT585277 FCYLPADPGRCLAYMPRFYYNPASNKCKEFIYGGCRGNANNFKTWDECRHTCV

VamKT585274 FCYLPADPGRCLAEMPRFYYNPASNKCKEFIYGGCRGNANNFKTWDECRHTCV

VbeJTGP01134452 ICHLPADPGICKAHTPRFYYDSASNKCKEFSFGRCPGNANNFKTRDQCRYTCV

VamKT585276 FCHLPADPGICKAHTPRFYYDSASNKCKQFSFGGCPGNANNSKTWDECRHTCV

OhaAZIM01010506b FCHLPPEHGPGLAIKYAYAYNPAANEFPRFSYGGCGGNANNFEAKGQCQRTCV

DpoS61886 DCKLPLRIGPCKRKIPSFYYKWKAKQCLPFDYSGCGGNANRFKTIEECRRTCV

OhaAZIM01010506a FCLLRPDRGPCEGNIRAFYYSPSSNSCQEFIYGGCQGNANRFKTNNECHHTCV

DsiAM411365 FCNLAPESGRCRGHLRRIYYNPDSNKCEVFFYGGCGGNDNNFETRKKCRQTCG

DsiAM411361 FCNLAPESGRCRGHLRRIYYNPDSNKCEVFFYGGCGGNDNNFETRKKCRQTCG

DsiAM411373 FCNLAPESGRCRGHLRRIYYNLESNKCKVFFYGGCGGNANNFETRDECRQTCG

DsiAM411369 FCNLAPESGRCRGHLRRIYYNLESNKCNVFFYGGCGGNDNNFETRDECRQTCG

DsiAM411362 FCNLAPESGRCRAHLRRIYYNLESNKCEVFFYGGCGGNDNNFSSWDECRHTCV

DsiAM411371 FCNLAPESGRCRGHLRRIYYNLESNKCEVFFYGGCGGNDNNFSTWDECRHTCV

DsiAM411370 FCNLAPESGRCRGHLRRIYYNLESNKCEVFFYGGCGGNDNNFSTRDECRHTCV

OaeGBIA01000036 FCYLPPETGICKAYVRAFYYNLTSNECQEFVYGGCVGNANRFETEDDCKDSCV

CmiJPMF01167077 FCYLPSETGPCKANVQAFYCNSASNNCEQFTYGGCHGNANNFETKNKCHYTCV

SfaGAHI01000009 YCDLPANPGPCRARITRFYYNSDSKQCEEFIYGGCHGNANNFETKDKCHYTCV

OhaAZIM01007380a FCYLPANPGPCRATITRFYYNSDSKQCEKFTYGGCHGNANNFETKDKCHYTCV

CmiJPMF01153046 LCYLPAEPGPCRGAITRFYYNLDSNKCQEFTYGGCRGNANNFKTKNKCHYTCV

VbeJTGP01134455a YCDLPADPGPCRGTIPRFYYNPASNKCQEFTYGGCKGNANNFKTKDKCHYTCV

VbeJTGP01134455b YCDLPADPGPCRGTITRFYYNSASKQCQEFTYGGCEGNANNFKTKDECHYTCV

PguGBIB01000048 LCHLPHEAGPCQVYIPRFYYSSTSNKCQQFIYGGCQGNANNFKTKDECHFTCV

OaeGBIA01000037 FCHLPQDGGPCLAYIPRFYYNSTSNECQEFIYGGCRGNANNFKTKDDCHFTCV

VbeJTGP01201326 FCHLPSDPGKCKAQIPRFYYNPASNQCQGFTYGGCGGNANNFKTWDECRHTCV

DsiJQ608454 FCYLPADPGECLAHMRSFYYNPASNQCQGFTYGGCGGNANNFETRDQCRHTCG

DsiAM411366 FCHLPADSGRCKAHIPRFYYNPASNQCQGFTYGGCGGNANNFETRDQCRHTCG

DruDQ365982 FCHLPVDSGICRAHIPRFYYNPASNQCQGFIYGGCEGNANNFETRDQCRHTCG

DruDQ365981 FCHLPVDSGICRAHIPRFYYNPASNQCQGFIYGGCGGNANNFETRDQCRHTCG

VbeJTGP01134462 FCYLPAETGLCEAIMPRFYYNPTSNECQKFIYGGCGGNANNFRTKDLCHYTCV

VbeJTGP01215779 FCSLPAETGPCKARMPRFYYNPASNQCQQFIYGGCKGNDNNFKTLDECRYTCV

OhaAZIM01007678 FCYLPAETGPCKAKMPRFYYNPASKQCEKFTYGGCKGNDNNFKTLDQCRYTCV

CmiJPMF01407963 FCYLPAETGPCKAKMPRFYYNQASNQCQPFIYGGCKGNDNNFKTLDQCRYTCV

PteAF402329 FCELPADIGPCDDFTGAFHYSPREHECIEFIYGGCKGNANNFNTQEECESTCA

PauAY626926 FCELPADTGPCRVGFPSFYYNPDEKKCLEFIYGGCQGNANNFITKEECESTCA

PteAF402324 FCELPADTGPCRVRFPSFYYNPDEKKCLEFIYGGCEGNANNFITKEECESTCA

OmiAY626930 LCELPADTGPCRVGFPSFYYNPDEKKCLEFIYGGCEGNANNFITKEECESTCA

OscAY626929 FCELPADTGPCRVGFPSFYYNPDEKKCLEFIYGGCEGNANNFITKEECESTCA

OscAY626928 FCELPADTGPCRVGFPSFYYNPDEKKCLEFIYGGCEGSANNFITKEECESTCA

SfaGAHI01000010 FCELPDDSGPCKGSFEAFYYNADQHKCLEFIYGGCDGNANNFKTIEECEHTCA

NscAY626932 FCELPADSGPCRGILHAFYYHPVHRTCLEFIYGGCYGNANNFKTIDECKRTCA

NscAY626933 FCELPADSGPCRGILHAFYYHPVHRTCLEFIYGGCYGNANNFKTIDECEPPCA

PpoAY626934 FCELPDDRGPCRGIFHAFYYNPDQRQCLEFIYGGCYGNANNFKTIDECERTCA

HbuGAHG01000008 FCELPDDSGPCRGIFHAFYYNPDQRQCLEFIYGGCYGNANNFKTIDECKRICA

BcaAY057888 FCNLLPEPGRCNAIVRAFYYNSRLRKCLEFPYGGCGGNANNFKTIDECQRTCA

BmuAM939782 FCNLLPEPGRCNAIVRAFYYNSRPRKCLEFPYGGCGGNANNFKTIEECQRTCA

DcoFJ752474 FCHLPADPGRCNALSEAFYYNPVQRKCLKFRYGGCKANANTFKTIDECKRTCA

PauAY626924 FCELPADPGPCNGLFQAFYYNPVQRTCLKFRYGGCKGNPNTFKTIEECKRTCA

ProAB576154 FCELPADPGPCNGLFQAFYYNPVQRKCLKFRYGGCKGNPNTFKTIEECKRTCA

PteAF402326 FCKLPAETGRCNAKIPRFYYNPRQHQCIEFLYGGCGGNANNFKTIKECESTCA

PmoGAHH01000050 FCELPAETGRCNAHITRFYYNPRQHQCIKFLYGGCGGNANNFKTIKECKSTCI

PmoGAHH01000051 FCELPAETGLCNAHNTRFYYNPRQHQCIKFLYGGCGGNANNFKTIEECKSTCI

VanGAHJ01000020 ICHLPANPGLCNGKFQAFYYNRIQRSCLMFMYGGCGGNANNFKTIDECKQTCV

CsqGAHB01000017 FCELPPDKGPCKGSFLAFHYHPVQQQCRQFTYGGCQGNPNNFKTIEECKSTCA

CsqGAHB01000019 FCELPADKGPCKGSFLAFHYHPVQQQCLQFLYGGCQGNPNNFETIEECKRTCA

CsqGAHB01000021 FCELPPDKGGCYGWLLAFYYNPVQHQCLQFIYGGCPGNANYFNTIEECKSTCA

ForGAHE01000028 FCELPADTGLCKRYIKAFYYSPGHHACLPFVYGGCPGNANNFKTIDECKRTCA

ForGAHE01000029 ICELPADRGLCKRYIKAFYYSPGHHACLPFIYGGCPGNANNFKTIDECKRTCA

HsiGAHF01000019 FCHLPHETGLCKRNIQAFYYDPVYHTCLKFIYGGCEGNANNFKTIDECKRTCA

HbuGAHG01000011 FCHLPHETGPCKRNTQAFYYNPVYHTCLKFIYGGCEGNSNNFKTIDECKRTCA

DcoFJ752473 FCHLPHETGPCKAKIQAFYYNPIYDTCLKFIYGGCEGNANNFKTMDECKRTCA

CsqGAHB01000018 FCHLPHDIGPCKAKFPAFYYDPVYHICGKFTYGGCQGNPNNFKTIEECKRTCA

PteAF402327 FCELPADTGSCKGNVPRFYYNADHHQCLKFIYGGCGGNANNFKTIEECKSTCA

VanGAHJ01000019 FCHLPHDTGPCKGIFQAFYYNPVQHQCREFIYGGCGGNANNFKTIDECKQTCV

VanGAHJ01000021 FCHLPHDTGPCKRNIQAFYYNPVQHQCREFIYGGCGGNPNNFKTIDECKQTCA

AweGAGZ01000018 FCHLPHDTGPCKGMFPAFYYHPGHRTCLQFIYGGCRGNANNFKTIEECKRICA

AweGAGZ01000016 FCHLPHDTGPCKGMFPAFYYHPGHRTCLQFIYGGCRGNPNNFKTIDECKRICA

AweGAGZ01000017 FCHLPHDTGPCKGMFPAFYYHPGHRTCLQFIYGGCRGNANNFKTIDECKRICA

EcuGAHD01000011 FCHLPYDTGPCKARFIAFYYNSVQRKCLEFVYGGCEGNANNFKTIDACKRTCA

AweGAGZ01000019 FCHLPADTGPCKANFLAFYYHPVHRKCLEFTYGGCEGNANNFKTIDECKRTCA

ProAB576155 FCHLPHDPGPCKGNFQAFYYHPVRRTCLEFIYGGCQGNPNNFKTIDECKRTCA

HsiGAHF01000018 FCELPADSGSCKGDFLAFYYNPNQHQCLEFSYGGCDGNDNNFKTIEECKRTCA

HsiGAHF01000017 FCELPADSGSCKGDFLAFYYNPDKHQCLEFSYGGCDGNDNNFKTIEECKRTCA

HbuGAHG01000010 FCELPADSGSCKGNFQAFYYNPVQHQCLEFIYGGCDGNANNFKTIDECKRTCA

DcoFJ752472 FCHLPADSGSCKGNFQAFYYHPVHRTCLEFIYGGCEGNANNFKTMDECKRTCA

ProAB576156 FCELPADSGSCKGNFQAFYYHPVHRTCLEFIYGGCEGNDNNFKTIDECKRTCA

PauAY626925 FCELPPDSGSCKGSFQAFYYHPVHRTCLEFIYGGCEGNDNNFKTIDECKRTCA

**Suppl. Fig. 5**. **Amino acid alignment of the second exon of snake venom Kunitz/BPTIs** including six *V. ammodytes*, nine *V. berus*, four *C. mitchellii* and four *O. hannah* as well as already annotated sequences and *Bos taurus* BPTI.


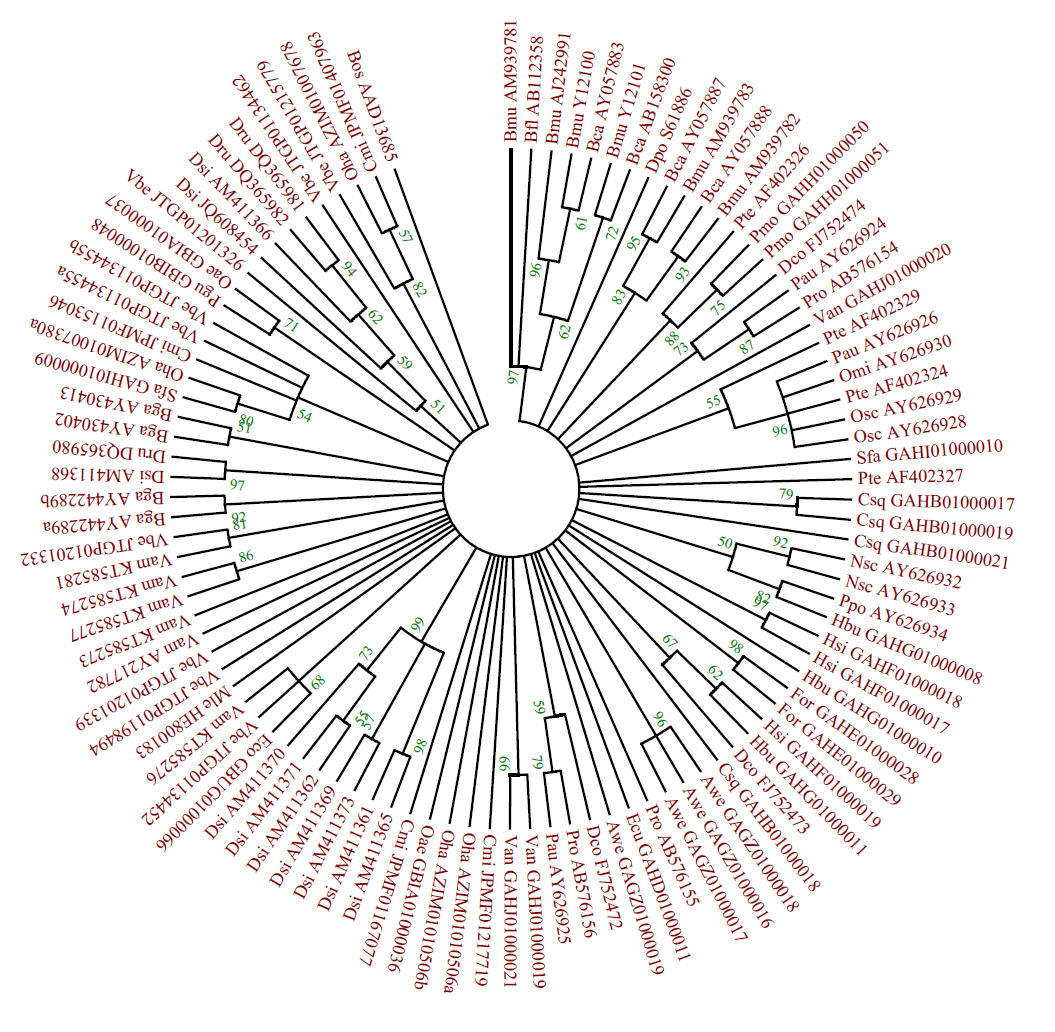


**Suppl. Fig. 6. Condensed ML tree of the snake venom Kunitz/BPTIs.** Phylogenetic analysis of the snake venom Kunitz/BPTI genes was conducted using the translated amino acid sequences of the exon 2. Branches corresponding to partitions reproduced in less than 50% bootstrap replicates are collapsed. As an outgroup, we used bovine BPTI. Phylogenetic analyses were performed with the program MEGA6^53^.

**Suppl. Table 5 Reference set of all representatives of the snake Kunitz/BPTI family**

|  | Species | Short Name/Accession Number | Protein | Selecton Viperidae | Selecton Elapidae | Selecton all | Phylogeny |
| --- | --- | --- | --- | --- | --- | --- | --- |
| 1 | *Acanthophis wellsi* | AweGAGZ01000016 | KP-Aca-6 |  |  |  |  |
| 2 | *Acanthophis wellsi* | AweGAGZ01000017 | KP-Aca-8 |  |  |  |  |
| 3 | *Acanthophis wellsi* | AweGAGZ01000018 | KP-Aca-9 |  |  |  |  |
| 4 | *Acanthophis wellsi* | AweGAGZ01000019 | KP-Aca-1 |  |  |  |  |
| 5 | *Bitis gabonica* | BgaAY442289a | two-Kunitz protease inhibitor |  |  |  |  |
| 6 | *Bitis gabonica* | BgaAY442289b | two-Kunitz protease inhibitor |  |  |  |  |
| 7 | *Bitis gabonica* | BgaAY430402 | Kunitz protease inhibitor 1 |  |  |  |  |
| 8 | *Bitis gabonica* | BgaAY430413 | Kunitz protease inhibitor 2 |  |  |  |  |
| 9 | *Bos taurus* | BosAAD13685 | BPTI |  |  |  |  |
| 10 | *Bungarus candidus* | BcaAY057883 | β-bungarotoxin B1 chain |  |  |  |  |
| 11 | *Bungarus candidus* | BcaAY057885 | β-bungarotoxin B2b chain |  |  |  |  |
| 12 | *Bungarus candidus* | BcaAB158300 | β-bungarotoxin B3 chain |  |  |  |  |
| 13 | *Bungarus candidus* | BcaAY057886 | Kunitz inhibitor a |  |  |  |  |
| 14 | *Bungarus candidus* | BcaAY057887 | Kunitz inhibitor b |  |  |  |  |
| 15 | *Bungarus candidus* | BcaAY057888 | Kunitz inhibitor c |  |  |  |  |
| 16 | *Bungarus flaviceps* | BflAB112358 | β-bungarotoxin B chain |  |  |  |  |
| 17 | *Bungarus multicinctus* | BmuY12100 | β-bungarotoxin B1 chain |  |  |  |  |
| 18 | *Bungarus multicinctus* | BmuAJ242991 | β-bungarotoxin B3 chain |  |  |  |  |
| 19 | *Bungarus multicinctus* | BmuY12101 | β-bungarotoxin B2 chain |  |  |  |  |
| 20 | *Bungarus multicinctus* | BmuAM939781 | PILP-1 |  |  |  |  |
| 21 | *Bungarus multicinctus* | BmuAM939782 | PILP-2 |  |  |  |  |
| 22 | *Bungarus multicinctus* | BmuAM939783 | PILP-3 |  |  |  |  |
| 23 | *Cacophis squamulosus* | CsqGAHB01000016 | KP-Cac-7 |  |  |  |  |
| 24 | *Cacophis squamulosus* | CsqGAHB01000017 | KP-Cac-8 |  |  |  |  |
| 25 | *Cacophis squamulosus* | CsqGAHB01000018 | KP-Cac-9 |  |  |  |  |
| 26 | *Cacophis squamulosus* | CsqGAHB01000019 | KP-Cac-10 |  |  |  |  |
| 27 | *Cacophis squamulosus* | CsqGAHB01000021 | KP-Cac-20 |  |  |  |  |
| 28 | *Crotalus mitchellii pyrrhus* | CmiJPMF01407963 | contig_411834 |  |  |  |  |
| 29 | *Crotalus mitchellii pyrrhus* | CmiJPMF01153046 | contig_153976 |  |  |  |  |
| 30 | *Crotalus mitchellii pyrrhus* | CmiJPMF01167077 | contig_168114 |  |  |  |  |
| 31 | *Crotalus mitchellii pyrrhus* | CmiJPMF01217719 | contig_219161 |  |  |  |  |
| 32 | *Daboia russellii russellii* | DruDQ365979 | Kunitz protease inhibitor-II |  |  |  |  |
| 33 | *Daboia russellii russellii* | DruDQ365980 | Kunitz protease inhibitor-III |  |  |  |  |
| 34 | *Daboia russellii russellii* | DruDQ365981 | Kunitz protease inhibitor-IV |  |  |  |  |
| 35 | *Daboia russellii russellii* | DruDQ365982 | Kunitz protease inhibitor-V |  |  |  |  |
| 36 | *Daboia siamensis* | DsiJQ608454 | DrKIn7880 protease inhibitor |  |  |  |  |
| 37 | *Daboia siamensis* | DsiAM411361 | trypsin inhibitor-1 China |  |  |  |  |
| 38 | *Daboia siamensis* | DsiAM411362 | trypsin inhibitor-2 China |  |  |  |  |
| 39 | *Daboia siamensis* | DsiAM411363 | trypsin inhibitor-3 China |  |  |  |  |
| 40 | *Daboia siamensis* | DsiAM411364 | trypsin inhibitor-4 China |  |  |  |  |
| 41 | *Daboia siamensis* | DsiAM411365 | trypsin inhibitor-5 China |  |  |  |  |
| 42 | *Daboia siamensis* | DsiAM411366 | trypsin inhibitor-6 China |  |  |  |  |
| 43 | *Daboia siamensis* | DsiAM411367 | trypsin inhibitor-7 China |  |  |  |  |
| 44 | *Daboia siamensis* | DsiAM411368 | trypsin inhibitor-1 Burma |  |  |  |  |
| 45 | *Daboia siamensis* | DsiAM411369 | trypsin inhibitor-2 Burma |  |  |  |  |
| 46 | *Daboia siamensis* | DsiAM411370 | trypsin inhibitor-3 Burma |  |  |  |  |
| 47 | *Daboia siamensis* | DsiAM411371 | trypsin inhibitor-4 Burma |  |  |  |  |
| 48 | *Daboia siamensis* | DsiAM411372 | trypsin inhibitor-5 Burma |  |  |  |  |
| 49 | *Daboia siamensis* | DsiAM411373 | trypsin inhibitor-6 Burma |  |  |  |  |
| 50 | *Daboia siamensis* | DsiAM411374 | trypsin inhibitor-7 Burma |  |  |  |  |
| 51 | *Dendroaspis polylepis* | DpoS61886 | dendrotoxin K |  |  |  |  |
| 52 | *Drysdalia coronoides* | DcoFJ752472 | serine protease inhibitor 161 |  |  |  |  |
| 53 | *Drysdalia coronoides* | DcoFJ752473 | serine protease inhibitor 87 |  |  |  |  |
| 54 | *Drysdalia coronoides* | DcoFJ752474 | serine protease inhibitor 18 |  |  |  |  |
| 55 | *Echiopsis curta* | EcuGAHD01000011 | KP-Ech-4 |  |  |  |  |
| 56 | *Echis coloratus* | EcoGBUG01000066 | Kunitz (ku-wap) |  |  |  |  |
| 57 | *Furina ornata* | ForGAHE01000028 | KP-Fur-8 |  |  |  |  |
| 58 | *Furina ornata* | ForGAHE01000029 | KP-Fur-18 |  |  |  |  |
| 59 | *Hemiaspis signata* | HsiGAHF01000017 | KP-Hem-8 |  |  |  |  |
| 60 | *Hemiaspis signata* | HsiGAHF01000018 | KP-Hem-9 |  |  |  |  |
| 61 | *Hemiaspis signata* | HsiGAHF01000019 | KP-Hem-12 |  |  |  |  |
| 62 | *Hoplocephalus bungaroides* | HbuGAHG01000008 | KP-Hop-3 |  |  |  |  |
| 63 | *Hoplocephalus bungaroides* | HbuGAHG01000010 | KP-Hop-9 |  |  |  |  |
| 64 | *Hoplocephalus bungaroides* | HbuGAHG01000011 | KP-Hop-11 |  |  |  |  |
| 65 | *Macrovipera lebetina transmediterranea* | MleHE800183 | PIVL |  |  |  |  |
| 66 | *Notechis scutatus scutatus* | NscAY626932 | tigerin-1 |  |  |  |  |
| 67 | *Notechis scutatus scutatus* | NscAY626933 | tigerin-2 |  |  |  |  |
| 68 | *Opheodrys aestivus* | OaeGBIA01000036 | Kunitz_SCG (ku-wap) |  |  |  |  |
| 69 | *Opheodrys aestivus* | OaeGBIA01000037 | Kunitz_SCG_SK (ku-wap) |  |  |  |  |
| 70 | *Ophiophagus hannah* | OhaAZIM01007678 | scaffold7682.1 |  |  |  |  |
| 71 | *Ophiophagus hannah* | OhaAZIM01007380a | scaffold7384.1 |  |  |  |  |
| 72 | *Ophiophagus hannah* | OhaAZIM01010506b | scaffold10511.1 |  |  |  |  |
| 73 | *Ophiophagus hannah* | OhaAZIM01010506a | scaffold10511.1 |  |  |  |  |
| 74 | *Oxyuranus microlepidotus* | OmiAY626930 | microlepidin-1 |  |  |  |  |
| 75 | *Oxyuranus scutellatus* | OscAY626928 | scutellin-1 |  |  |  |  |
| 76 | *Oxyuranus scutellatus* | OscAY626929 | scutellin-2 |  |  |  |  |
| 77 | *Pantherophis guttatus* | PguGBIB01000048 | Kunitz_SK (ku-wap) |  |  |  |  |
| 78 | *Pseudechis australis* | PauAY626924 | mulgin-1 |  |  |  |  |
| 79 | *Pseudechis australis* | PauAY626925 | mulgin-2 |  |  |  |  |
| 80 | *Pseudechis australis* | PauAY626926 | mulgin-3 |  |  |  |  |
| 81 | *Pseudechis porphyriacus* | PpoAY626934 | blackelin |  |  |  |  |
| 82 | *Pseudechis rossignolii* | ProAB576154 | Pr-mulgin 1 |  |  |  |  |
| 83 | *Pseudechis rossignolii* | ProAB576155 | Pr-mulgin 2 |  |  |  |  |
| 84 | *Pseudechis rossignolii* | ProAB576156 | Pr-mulgin 3 |  |  |  |  |
| 85 | *Pseudonaja modesta* | PmoGAHH01000050 | KP-Pse-5 |  |  |  |  |
| 86 | *Pseudonaja modesta* | PmoGAHH01000051 | KP-Pse-7 |  |  |  |  |
| 87 | *Pseudonaja textilis textilis* | PteAF402324 | textilinin-1 |  |  |  |  |
| 88 | *Pseudonaja textilis textilis* | PteAF402326 | textilinin-3 |  |  |  |  |
| 89 | *Pseudonaja textilis textilis* | PteAF402327 | textilinin-4 |  |  |  |  |
| 90 | *Pseudonaja textilis textilis* | PteAF402328 | textilinin-5 |  |  |  |  |
| 91 | *Pseudonaja textilis textilis* | PteAF402329 | textilinin-6 |  |  |  |  |
| 92 | *Suta fasciata* | SfaGAHI01000009 | KP-Sut-1 |  |  |  |  |
| 93 | *Suta fasciata* | SfaGAHI01000010 | KP-Sut-2 |  |  |  |  |
| 94 | *Vermicella annulata* | VanGAHJ01000018 | KP-Ver-2 |  |  |  |  |
| 95 | *Vermicella annulata* | VanGAHJ01000019 | KP-Ver-3 |  |  |  |  |
| 96 | *Vermicella annulata* | VanGAHJ01000020 | KP-Ver-5 |  |  |  |  |
| 97 | *Vermicella annulata* | VanGAHJ01000021 | KP-Ver-13 |  |  |  |  |
| 98 | *Vipera ammodytes ammodytes* | VamAY217781 | Trypsin inhibitor |  |  |  |  |
| 99 | *Vipera ammodytes ammodytes* | VamAY217782 | Chymotrypsin inhibitor |  |  |  |  |
| 100 | *Vipera ammodytes ammodytes* | VamKT585273 | Kunitz/BPTI inhibitor-1 |  |  |  |  |
| 101 | *Vipera ammodytes ammodytes* | VamKT585274 | Kunitz/BPTI inhibitor-2 |  |  |  |  |
| 102 | *Vipera ammodytes ammodytes* | VamKT585275 | Kunitz/BPTI inhibitor-3 |  |  |  |  |
| 103 | *Vipera ammodytes ammodytes* | VamKT585276 | Kunitz/BPTI inhibitor-4 |  |  |  |  |
| 104 | *Vipera ammodytes ammodytes* | VamKT585277 | Kunitz/BPTI inhibitor-5 |  |  |  |  |
| 105 | *Vipera ammodytes ammodytes* | VamKT585278 | Kunitz/BPTI inhibitor-6 |  |  |  |  |
| 106 | *Vipera ammodytes ammodytes* | VamKT585279 | Kunitz/BPTI inhibitor-7 |  |  |  |  |
| 107 | *Vipera ammodytes ammodytes* | VamKT585280 | Kunitz/BPTI inhibitor-8 |  |  |  |  |
| 108 | *Vipera ammodytes ammodytes* | VamKT585281 | Kunitz/BPTI inhibitor-9 |  |  |  |  |
| 109 | *Vipera berus berus* | VbeJTGP01215779 | contig_215779 |  |  |  |  |
| 110 | *Vipera berus berus* | VbeJTGP01134462 | contig_134462 |  |  |  |  |
| 111 | *Vipera berus berus* | VbeJTGP01134455a | contig_134455 |  |  |  |  |
| 112 | *Vipera berus berus* | VbeJTGP01134455b | contig_134455 |  |  |  |  |
| 113 | *Vipera berus berus* | VbeJTGP01201326 | contig_201326 |  |  |  |  |
| 114 | *Vipera berus berus* | VbeJTGP01134452 | contig_134452 |  |  |  |  |
| 115 | *Vipera berus berus* | VbeJTGP01201332 | contig_201332 |  |  |  |  |
| 116 | *Vipera berus berus* | VbeJTGP01198494 | contig_198494 |  |  |  |  |
| 117 | *Vipera berus berus* | VbeJTGP01201339 | contig_201339 |  |  |  |  |
|  |  |  |  | 39 | 17 | 100 | 101 |
